# Military Nursing in the Brazilian Army (1992–2020): perspectives of nursing officers in light of Bourdieu*

**DOI:** 10.1590/1980-220X-REEUSP-2025-0234en

**Published:** 2025-11-28

**Authors:** Fabrícia Conceição de Carvalho, Maria Itayra Padilha, Fernanda Batista Oliveira Santos, Paulo Joaquim Pina Queirós, Vanessa Ribeiro Neves

**Affiliations:** 1 Exército Brasileiro, Hospital Militar de Área de São Paulo, São Paulo, SP, Brazil.; 2 Universidade Federal Santa Catarina, Departamento de Enfermagem e do Programa de Pós-Graduação em Enfermagem, Florianópolis, SC, Brazil.; 3 Universidade Federal de Minas Gerais, Departamento de Enfermagem Básica, Belo Horizonte, MG, Brazil.; 4 Escola Superior de Enfermagem, Departamento de Enfermagem Fundamental, Coimbra, Portugal.; 5 Universidade Federal de São Paulo, Departamento de Serviços de Administração em saúde e em Enfermagem, São Paulo, SP, Brazil.

**Keywords:** History of Nursing, Nursing, Military Personnel, Military Nursing, Military Health Services

## Abstract

**Objective:**

To analyze the perspectives of Nursing officers regarding Military Nursing in the Brazilian Army in light of Bourdieu, from 1992 to 2020.

**Method:**

Study of historical-social interest, with a qualitative approach to Thematic Oral History. Fifteen nursing officers who worked in health services or in the Brazilian Army Health Fund were interviewed. Data were transcribed, transcreated, categorized, the content analyzed according to Minayo, and the findings interpreted in light of Bourdieu’s Theory of the Social World.

**Results:**

The category “Nursing and the Brazilian Army: the gathering of hierarchies” and the subcategories “Military values”, “Gender in hierarchical military relations” and “From being a civilian to being a military person” were identified. From the participants’ perspective, Military Nursing presents influences from military, gender, and intellectual capital of Nursing on its professional identity.

**Conclusion:**

The trajectory of Military Nursing maintained today by the Brazilian Army Nursing officers provides perspectives influenced by the volume of capital in hierarchical military relations as well as the symbolic profit of Military Nursing.

## INTRODUCTION

The Crimean War (1853–1856) was the stage for the consolidation of the existence of Modern Nursing, which had nurse Florence Nightingale as the protagonist of the transformation of care, based on her legacy in field hospitals. This transformation enabled the dissemination of values present in the military practice by Nightingale and her team’s scenario of action to the practice of Nursing: hierarchy and discipline^(1)^. Nursing and the military institution have a historical connection, whose environment is a place of contemporary nursing practice in the world and in Brazil^(2)^. Thus, Nursing in war contexts specializes as Military Nursing, which, although not formally recognized in Brazil, is conventionally identified in practice by its military nurses.

The rigid organizational culture present in the military institution is materialized in the division of roles for the fulfillment of orders, while limiting individual autonomy, as no modifying idea or suggestion remains restricted to its holder, being always re-evaluated by the higher leadership to maintain the typical organizational *status quo* of obedience, discipline, and hierarchy^(3)^. In this regard, during her work in the field hospitals in Crimea, even though Nightingale strictly followed the rules, she suffered from this hierarchical rigidity, linked to the cultural resistance to her female presence on the part of the medical officers, as she and the other nurses could not be present in the pavilions where these officers were. However, as she was faithful to the military formalities, she managed to be accepted in relations with the military medical team, and gained popularity through her work, which, in the health and military aspect, contributed to the reduction of the mortality rate and consequent minimization of soldier casualties, favoring the permanence of the troops in the battle front^(2)^.

In this Nursing historical feat, it is possible to identify facts that materialize the concepts of *habitus* and capital in the power relations typical of a military environment from the perspective of the French philosopher and sociologist Pierre Bourdieu^(4)^. The *habitus* is an embodied disposition that manifests itself in the lifestyle, attitudes, and way of being of individuals. In the military context, it reflects the internalization of military capital, legitimized by ranks or progressions. These positions, depending on their volume and prestige, represent concrete opportunities for obtaining symbolic profits, derived from recognition within the institutional hierarchy.

Capital is synonymous with power, as it reflects the position of an agent (individual or institution) based on their characteristics in the social system. Thus, capital can be economic (financial resources), cultural (knowledge, beliefs and values), social (representation in the group), among others. The holder of capital will have, as a consequence, symbolic profit and power in social relations^(5)^.

In this sense, Florence Nightingale’s observance of the formalities present in the military hierarchy represents the inculcation of a *habitus* which enabled the acquisition of the capital required to belong to the military social group. This capital is part of the military cultural capital which, associated with military ranks or progressions, increases its volume to legitimize the symbolic profit of recognition in the power relations of the military hierarchical circle. Nightingale, coming from a civilian background, did not have the military capital resulting from patents, but her military *habitus*, acquired and associated with work in the Crimean War, formed cultural capitals that brought power for representation and meaning in Nursing that went beyond the military field, becoming a model for the professionalization of Nursing care throughout the world^(6)^. Therefore, the military environment was for Nursing what Nursing was for the military environment: a symbolic profit achieved through the performance of nurses on the battlefield, which led to the gathering of hierarchies represented by military values common to both professions, Military and Nursing: obedience, discipline and hierarchy, which gave rise to Military Nursing^(7)^.

Military Nursing has been present in the Brazilian Army since 1944, when the Reserve Force Nurses staff of the Brazilian Expeditionary Force (*FEB*) was created during the Second World War. With the end of the war, a 12-year gap was created and, from 1957 onwards, the FEB reserve nurses would close this gap with their reinclusion as active officers in this same staff^(8)^. With the retirement of nursing officers, a new gap in the Army Nursing emerged and remained for 35 years, being filled in 1992, when Nursing returned to its ranks through the Complementary Officers’ Staff (*QCO*), complemented by the technical support of Nursing also present in another officer personnel created in 2018: Temporary Technical Officer (*OTT*) Staff^(9,10)^. These staffs are for administrative purposes and are intended to support the Army’s military specialties in administrative-military matters, present in the arms, staffs and services, such as the Health Service. Thus, Nursing is present in the administrative personnel of the Brazilian Army to assist in areas of interest to the institution for combat. In this regard, particularly in the Health Service, Military Nursing acts in the care aspect as support for care practices, either through direct action in clinical-surgical units in military hospitals, providing Nursing care according to the client profile, or through the management and administration of the Nursing service, or through continuing education aimed at military Nursing personnel or health education for users of the military health system, or through indirect action, in administrative-hospital support sectors such as warehouses, laboratories, bidding and contracts sections or even in medical specialty outpatient clinics. They also work in the management of healthcare contracts and in the auditing of these contracts in the prospective, concurrent, and retrospective phases of their execution.

Still in that service, Military Nursing also works in the operational aspect, providing pre-hospital care and applying knowledge in basic and advanced life support. Therefore, Military Nursing operates in the Brazilian Army according to the institutional needs and interests of the Military Public Administration, providing services according to health demands (Health Service) and administrative demands of other military specialties (arms, personnel, and services), all of which are cumulative.

This accumulation can, at times, alienate the Nursing professional identity by superimposing the military perspective on the fulfillment of orders, which distances Nursing from its professional essence in health and its leading role in care.

The professional practice of Military Nursing in the Brazilian Army presents a reality that deviates from the usual reality of practice in the civilian environment, where Nursing only works in its area of training and specialty, which can contribute to a weakening of the image of Nursing and to the removal of the representation of the professional class.

Therefore, research into Military Nursing is important for understanding this reality by identifying the perceptions of military nurses who work in this secular career, to allow the understanding of the meanings of Nursing care provided in a military environment. Furthermore, Military Nursing is an area with a shortage of national bibliographic production^(11)^, so that studies like this can mitigate this gap and provide greater clarity about the military world as a space for the professional practice of Nursing. The aim of this study is to present the experience of military professional practice from the perspective of Nursing officers belonging to the *QCO* and *OTT* of the Brazilian Army so that it can be contextualized with the historicity experienced by Florence Nightingale in a scenario of power relations in the military environment.

The justification for this study is present in the contribution it can bring to the profession of Military Nursing in Brazil and in the world regarding knowledge of the military area for professional practice, which still has limits regarding the presence of the female gender, and which, despite being a historical field of the professional presence of Nursing, is still a recent field, lacking scientific innovation in Nursing, requiring advances.

Nursing as a predominantly female profession^(11)^, which arises from the hierarchy of work, reveals, in itself, limits in professional interrelationships, which are increased in limiting spaces of power, such as the military space, favoring inequality and symbolic gender violence, in addition to weakening the sense of group and professional identity in Nursing^(5,11)^.

This study aims to identify the perceptions of Nursing officers about Military Nursing in the Brazilian Army from 1992 to 2020.

## METHOD

Descriptive study, of historical-social interest, with a qualitative approach to Thematic Oral History^(12)^. The script of the *Consolidated Criteria for Reporting Qualitative Research* (COREQ)^(13)^ was followed in the method development.

The time frame of this research begins in 1992, as it was the milestone for the re-entry of the Nursing professional as a career officer in the Complementary Officers’ Staff (*Q*CO), and 2020 as the final cut, due to the entry of the first Nursing officers into the Temporary Technical Officer Staff (*OTT*), in 2018, despite having been created in 2002 through Decree No. 4,502^(14)^.

This research was submitted for consideration by the Human Research Ethics Committee (*CEP*) of the Universidade Federal de São Paulo (*UNIFESP*) with presentation certificate (CAAE) 54969421.7.0000.5505 and approved by Opinion No. 5,584,693.

Data collection was carried out using a semi-structured interview script applied to participants identified as Military Officers 01 to 15, previously selected based on the inclusion criteria defined for the study. The questionnaire contains questions of interest about leadership and career motivation, and participants were given the opportunity to talk about their professional experiences, the pertinent data of which were considered in the study.

The inclusion criteria for this study were: active or paid reserve nursing officers (retired R1), members of the *QCO*, and active or unpaid reserve nursing officers (temporary officers on voluntary leave or after completing 8 years of service R2), members of the *OTT* who, from 1992 to 2020, held a coordination/management position in a hospital sector of the Army Health Service or a coordination/management position in the Army Health Fund. Regarding the exclusion criteria: active or reserve Nursing officers (R1), members of the *QCO*, active or reserve Nursing officers (R2), members of the OTT who, in the time frame of this study, only held a coordination/management position at a Garrison or Military Battalion Medical Unit. These spaces were not considered in the study, as the role of the *QCO* or *OTT* nurse officer is minimal or non-existent, regarding leadership activities in health management or direct care. To preserve the anonymity of the record, the participating military personnel were represented by the following coding: military 01, military 02 and so on up to military 15.

According to data from the Brazilian Army Health System obtained in 2023^(15)^, the number of *QCO* nurses in the country totaled 629, while the number of *OTT* nurses was not disclosed. These incomplete data, associated with the dispersion of military nurses working in the 12 Military Regions, from the north to the south of Brazil, hindered the sample size estimation for sending invitations for the participation in the study. Thus, data collection, which began in 2023, occurred as follows: the contact channel for sending an invitation text to participate in the study was *WhatsApp*, given that the Brazilian Army’s communication channels are exclusive to matters of interest to the Ministry of Defense related to territorial defense and humanitarian missions. Thus, groups of *WhatsApp* of military nurses from the *QCO* and *OTT* personnel were notified to voluntarily participate in the study. The study was also publicized to Nursing officers who worked in the same military unit as the researcher and to whom, upon showing interest in participating, an invitation text was sent. The principal investigator analyzed the professional profile of interested parties to verify whether they met the study’s inclusion criteria and excluded those who did not meet them. In turn, those who met the inclusion criteria were suggested to invite more Nursing officers with whom they had contact (snowball). These also had their profile analyzed in terms of the study’s inclusion criteria. Finally, the Free and Informed Consent Form (FICF) was sent by email to the selected participants and, after being answered and signed, was sent back to the researcher’s email.

With the data collected, the process of transcription, transcreation, categorization, and content analysis was conducted according to Minayo’s model^(16)^. Data interpretation was done according to Pierre Bourdieu’s Theory of the Social World^(17)^.

This research has data from the dissertation “Identidade e perspectivas da Enfermagem Militar Brasileira: uma análise historiográfica (1992-2020)”, which can be obtained through the repository https://repositorio.unifesp.br/items/49880e2d-d9d3-4420-a7e6-53b1c60f43fc ^1^.

In this text, the content of the most significant interviews was selected for the analysis of the subcategories “military values”, “gender in hierarchical military relations”, “from being civilian to being military” in light of Pierre Bourdieu, which culminated in the selection of the statements of military personnel 02, 03, 04, 06, 08 and 10 of the 15 military personnel participating in the referenced research.

## RESULTS

After meeting the inclusion criteria, 15 participants remained, 11 women and four men. Among the women, four were *QCO* Nursing officers, one an R1 officer, and the other three from active duty, and seven were *OTT* Nursing officers: one from active duty and six R2 officers. Among the men, one was a *QCO* Nursing Officer, being R1, and the other three were *OTT* Nursing Officers, all on active duty. Ages ranged from 32 to 39 years for active duty *OTT* and R2 officers, 33 years for active duty *QCO* officers, and 52 to 59 years for R1 *QCO* officers. The length of professional service was two to five years for active *OTT* officers and up to eight years for *OTT* R2 officers. For *QCO* officers, the length of professional service was nine years for active officers and 17 to 33 years for R1 officers.

Based on the analysis of the objective and information from each interview, the pertinent hypothesis shown in Chart 1 was developed. When performing content analysis^(16)^, 12 recording units were identified within the context unit: “Capitals of the Nursing profession and the military profession”. In this context unit, the three subcategories, “Military values”, “Gender in hierarchical military relations” and “From being a civilian to being a military person”, were identified as meanings of the participants’ speeches, and resulted in the category “Nursing and the Brazilian Army: the gathering of hierarchies”.

**Chart 1:** Hypothesis outline – Capitals. São Paulo, SP, Brazil, 2024.

Hypothesis: Capitals			
**Category**	Nursing and the Brazilian Army: the gathering of hierarchies.		
**Subcategories**	Military values	Gender in hierarchical military relations	From being a civilian to being a military man
**Context unit**	Capitals of the Nursing profession and the military profession.		
**Registration units**	Resilience; Communication; Team spirit; Leadership; Nursing knowledge; Updating; Military knowledge; Gender; Problem solving; Conflict management; Proactivity; Public administration.		

***Military values***

*From everything I’ve experienced, I realize that to be a Nursing officer in the Brazilian Army, you need technical knowledge, obedience to hierarchy and discipline, knowing how to position yourself and communicate, having resilience, patience, teamwork skills, and the humility to accept what cannot be changed.* **(Military 2).**

*[...] the nursing officer must demonstrate skills in: technical knowledge in the area of activity, improvement through scientific updating, managing conflicts, having authority, autonomy, active listening, treating everyone with respect. It is necessary to have fair conduct in interprofessional relationships, treating unequals unequally [...].* (**Military 8).**

*[...] I believe that Military Nursing is viewed with great admiration due to the selflessness of personal life [...] I believe that Military Nursing is an important area of Brazilian nursing because it has all these aspects: the defense of national sovereignty, assistance to the population, and readiness to act in different places when necessary and designated.* (**Military 10).**

***Gender in hierarchical military relations***

*I was the only nurse in this unit and the person who delegated to me was a female Captain physician, who was the head of the Medical Unit. [...] When I showed her the needs of the sector, when I told her “we need to do this, we need to do that”, she paid little attention, at the same time that she wanted me to make changes in the service, she limited me in implementing these changes. [...].* **(Military 3).**

*In my experiences as a nursing officer, I could perceive the astonishment and surprise when some people knew my routine in some military activities, or when I presented my war name* ^2^ *, whose surname suggested that I might be a man. Some even questioned my feminine ability to command men.* **(Military 4).**

*I entered as a nurse earning based on a rank, and not because I was a woman, because we see many jobs in the civil service where there is a devaluation of women’s salaries. Nursing, being an essentially female profession, has a salary disparity compared to the medical profession, which is four or five times greater. So, in the military career, this does not happen because Nursing will earn by rank [...] I had challenges in the exercise of the profession, but it was an excellent experience [...].* **(Military 8).**

***From being a civilian to being a military officer***

*I graduated in Nursing in 2010, shortly after I worked in Epidemiology for two years and then [...] I worked at the Municipal Hospital for four years. I’ve always been fascinated by the Army [...] I was thrilled to see women in uniform, but I had no idea how to access work in the Armed Forces until a friend married to a military man told me about the 2014 selection process, which I took and was approved [...] Before joining the Army, I didn’t know what Military Nursing was like [...] The first few years were very good, I managed to do good work, have a lot of autonomy in my actions, but throughout my career I had a lot of difficulty [...] in which I became demotivated with the loss of autonomy and with the prevalence of the military part over the Nursing part.* (**Military 2**).

*I graduated from the Universidade Federal de of São Paulo in 2010. In 2011, I worked at Hospital São Paulo in the pediatric surgery unit and in the pediatric bone marrow transplant unit at GRAAC. At the end of 2014, I left GRAACC and started working in a clinic [...] I left that clinic to join the Army ranks in 2018 through the temporary technical officer selection process [...] the word I would use to define what it’s like to work in the Army would be challenge [...] it’s a completely different world [...] I realized that the military hospital does not have clear rules like a civilian hospital, which made me question my continued presence at the institution and my autonomy as a nurse. [...] In addition to all these institutional difficulties in our work routine, I have a concern about the role of the nurse here: whenever I see a nurse in functions that do not need to be performed by him/her [...] it bothers me [...].* (**Military 6).**

## DISCUSSION

Nursing builds its professional history in war fronts, establishing a professional identity that in the military field appropriates a specificity regarding military values, experience of achievements, advances and challenges, mainly regarding the insertion of the female gender in hierarchical military relations, in addition to the cultural shock from the entry of a civilian professional *habitus* trying to shape himself to belong in the military field^(3,7,17,18)^. In this regard, military values, the female gender in hierarchical relationships and the civilian professional expertise of Nursing, entering a military environment, will be discussed to enable knowledge and reflection on the reality of military Nursing practice.

Military Nursing has the professional particularity of an identity that considers values extrinsic to Nursing, which are the military values. Regarding military values, technical-professional improvement was present in the speeches as relevant to military nursing practice. Faith in the mission, which is synonymous with resilience in the face of adversity and challenges experienced in a military career, and patriotism that exalts service to the Fatherland were also identified. Love for the profession was evident in the speeches about the dedication and selflessness required in a military career. In turn, esprit de corps was identified when the participants referred to teamwork as a factor to be considered by the Nursing officer. These values provide the opportunity to characterize symbolic capitals^(17,18)^ present in the professional identity of Military Nursing. According to Bourdieu^(18)^, the *habitus* prepares the individual for the social space by providing him with skills that characterize him for belonging in the environment. Therefore, when a Nursing professional with *habitus* from the civil health environment enters the military environment, he/she is prepared for the acquisition of the military *habitus* identified by the uniform attire, the strictness of the hair and beard style, the absence of adornments, makeup and the use of discreet nail polish for women, also the use of medals when decorated for some extraordinary feat, the intonation of the voice for command orders in situations of military activities, the synchronized body movements and muscle tone in military graduations. Thus, the transition from a civilian nursing career to a military career promotes a personal transformation, which results in military capital associated with the intellectual capital that differentiates Military Nursing for symbolic gain in the space of military professional activity.

Regarding gender in hierarchical military relations, the view of each Nursing officer regarding the female gender in the Brazilian Army was noticeable. In particular, for nursing officers participating in the study, the military career offers better working conditions compared to the civilian environment, in terms of equality of professional practice and salary, as this equivalence is based on hierarchical rank. Although there is inequality in female representation in high-ranking positions, it is observed that few women reach the rank of colonel. Furthermore, at the time of this study, no woman had risen to the rank of general.

All of this externalizes the difficulty of accepting the female presence by the military group, which connotes a cultural resistance that still exists, such as that which occurred on the battlefield with Florence Nightingale in 1853^(2)^.

There were also reports of symbolic violence among women themselves, when in the same professional-military space, whether as a leader or as a team member, there is no female support in this environment. In this regard, it is important to discuss the concept of symbolic violence from Bourdieu’s perspective. This is a form of invisible violence that is imposed in a subjugation-submission relationship, in which recognition and complicity in violence are present components of the dominator-dominated relationship. Language, gestures, social exclusion, symbols and forms of communication or knowledge are examples of symbolic domination that are established through a process sustained by the existence and reinforcement of thoughts and predispositions aligned with imposed structures^(19)^.

The State, as an example of a hegemonic organizational institution, in addition to the family and the church, uses agents such as Military Forces to establish social and moral conformity. In turn, these agents endowed with a hierarchical organizational relationship appropriate the means, materials and symbolisms that guarantee the conformity of logic and morality to the ways of understanding and constructing the world according to dominant interests, and this will result in the symbolic violence that is so naturalized and intrinsic. The female gender, as a socio-group historically subjugated by male domination, perpetuates the unequal gender relationship in densely hierarchical spaces of power such as Military Forces^(19,20)^. Therefore, the occupation of leadership and representation in high-level military power, today, is mostly limited to the male group, with low female representation in the military positions of colonel and, for generals, currently non-existent for women in the Brazilian Army.

Moreover, military capital tied to position in the hierarchy favors the power struggle in the field, in which the volume of this capital influences the legitimization of agents. Associated with this, it is noted that the symbolic capital of gender, not yet institutionally validated for belonging and acceptance by the male military group, expresses an unfavorable social perception among women for not having this capital and the validation of their presence^(5,19,20,21,22,23,24, 25)^. Therefore, it is through military capital that women try to mark their presence in the military field and achieve power.

In the view of a male officer interviewed, women have advanced in their presence in the military scenario, since their participation in wars, increasing female representation in male military spaces.

In fact, the female gender made progress when it dared to occupy traditionally male spaces, such as the military space, favoring not only the symbolic effect of social redefinition of the image of women but also a symbolic gain for the professional space conquered by female professions such as Nursing^(3,5,6)^. However, there are still challenges to be faced regarding the cultural resistance of their presence for professional advancement in certain military positions, which, in the case of the Brazilian Army, relates to high military leadership positions of general officer, as they are not yet available to women, nor for Nursing.

The gender symbolic capital^(18)^ emerges as an additional volume to military capital in hierarchical military relations in which, specifically in the Brazilian Army, the capital represented in the profession still repels forces related to other agents and their capital, such as the male, as it is a quantitative and qualitative minority in the military social circle^(5,20,21,22,23,24,25)^.

In the logic of power relations^(17,18)^ existing in any social group, in the military group the female gender present in Military Nursing faces struggles that are intensified by the symbolic capital of gender and Nursing intellectuals, whose legitimacy is weakened in the military hierarchical cycle, which is densely masculine and doctor-centered. Thus, Military Nursing in hierarchical military relations presents similarities with the symbolic violence experienced by Florence Nightingale in the Crimean War, in the 19th century, although it is necessary to relativize this similarity, considering that, at that time, women were not expected to perform qualified professional roles, much less hold a prominent hierarchical position within the military force^(1,2)^.

Currently, Military Nursing is predominantly female (symbolic capital) and also undervalued, as it is in administrative positions and not in the Health Service, lacking the power for institutional validation and the possibility of transforming this underrepresentation through Military Nursing leadership in the top echelons of Health Service officers: the hierarchical rank of general officer.

There is a dissociation between Care Nursing and Administrative Nursing, with a prioritization of military practice, promoting a focus on administrative tasks and military activities, with the alienation of their complete identity as a health professional. The consequence is the institutional invalidation of the health profession for belonging to the Armed Forces Health Service beyond a military professional identity, which favors the perpetuation of the current care model and medical hegemonic domination^(23)^.

Regarding the professional trajectory of Nursing officers, the majority came from civil society with a *habitus* different from the military, which generates many conflicts when performing Military Nursing^(3)^. With the exception of some military personnel participating in the study, who already had military *habitus* when entering the ranks of the Army to naturalize the specificities of the practice of Military Nursing, the other interviewees showed explicit or implicit frustration, observed in the facial expression during the interviews, in the face of the professional practice of Military Nursing of demanding self-denial of their professional identity in Nursing to comply with military orders based on the organizational principles of hierarchy and discipline^(19)^.

Regarding the symbolic power in the practice of Military Nursing in the Brazilian Army, the military hierarchy validates it for institutional recognition of Nursing as a military agent, while weakening the professional identity of Nursing due to the predominance of military identity through the determination of Nursing to carry out actions not related to health practice, but to the practice of military work and which undermine the image of Nursing as a care profession^(9,10).^

From the above, it was identified that from Civil Nursing to Military Nursing there is a cultural transformation of inculcation of military *habitus* to train Nursing officers, which, for most of the officers participating in this study, is a source of strangeness and frustration in military professional practice, with the imposition of this *habitus* to the Nursing professional identity.

Consequently, Nursing officers, in compliance with military orders that distance the officer from the professional practice of Nursing — as these orders represent the military *habitus* —, have their symbolic power in Nursing limited and their professional identity as Military Nursing weakened. This means that the power relations existing in the military social group present opposing forces that impede the existence of Nursing in the Brazilian Army, revealing the perpetuation of resistance to the presence of Nursing in the military scenario in Brazil, since the presence of nurse officers in the Brazilian Expeditionary Force.

Thus, this study presented as a limitation the in-person access to participants for data collection, since they were all dispersed territorially, which justified the collection of information for voluntary participation, after dissemination of the invitation text in the *WhatsApp group*, via email sent, *Google Forms* for the participation of participants’ guests (snowball), use of the platform *Zoom* to conduct the interviews.

The contribution of this study is to be a source of data on Military Nursing in Brazil and worldwide, as it is an area that lacks more publications to fill bibliographic gaps on the professional identity of Military Nursing. Furthermore, it instigates reflection and critical thinking about the military professional group of Nursing and the capitals as the core of the symbolic power of Nursing in the military scenario, favoring the clarification of the logic of power that perpetuates the barrier of invalidation of Nursing as a care profession in the Brazilian Army.

## CONCLUSION

Although Nursing is a profession whose history is narrated through military history, which, in turn, has been present in the history of the Nursing profession since 1853, Military Nursing faces challenges of recognition and belonging in the Armed Forces. In Brazil, because the Army is a traditional institution that values a culture of valuing heroic deeds and maintaining beliefs that shape professional military practice, this hinders transformations in organizational work models, which in the case of military health have a model materialized by the preservation of care traditionally centered on the medical officer.

As the Brazilian Army’s military health care model is medical-centric and focused on interventionist approaches, in a pragmatic perspective typical of the military sphere, Military Nursing is still disregarded in its professional health role. Hence, the symbolic power of Military Nursing is linked to hierarchical rank and not to its knowledge in health; when there is an overlap of rank (military capital) in a relationship in which there is a nurse-captain and a doctor-lieutenant, the captain will be given voice and legitimacy due to the military capital granting her power in the Armed Forces. However, when, in the military relationship, there is the same capital in dispute for power, lieutenant-nurse *versus* lieutenant-doctor, or the predominance of military-doctor capital in the captain-doctor relationship *versus* nurse lieutenant, there will be the removal of the symbolic power of Nursing to validate the military capital and medical capital recognized in the military field.

The results of this study revealed that the military pillars of hierarchy and discipline, values of the military profession, guided the Nursing profession since the Crimean War, as well as the Military Nursing profession, which enabled its professional identity in the military field. The latter has the symbolic capital of gender, the military capital, and the intellectual capital of Nursing, which may or may not represent the power that legitimizes an identity according to the perception and actions of Nursing officers in the military hierarchical circle.

The trajectory of Military Nursing maintained today by the Brazilian Army Nursing officers provides practice perspectives which are influenced by the weight and volume of capital in hierarchical military relations and that validated the professional identity of the Military Nursing in the Brazilian Army. In this regard, it can be inferred that from 1992 to 2020, when Nursing was allowed to operate in the Brazilian Army, through the Complementary Officers Staff and the Temporary Technical Officer Staff, there was a symbolic gain for Nursing and for Brazilian women who, in a space of military power, achieved equal pay and equal professional practice, even though inequality in the occupation of high leadership positions remains today.

As a symbolic effect of the presence of Nursing in the Brazilian Army, a military identity was given to the Nursing profession operating in this scenario, and contributed to a new specialty: Military Nursing.

## Data Availability

The data supporting the findings of this study are available in the UNIFESP Institutional Repository at the following link: https://repositorio.unifesp.br/items/49880e2d-d9d3-4420-a7e6-53b1c60f43fc.
