# Supplementary material for: Military Nursing in the Brazilian Army (1992–2020): perspectives of nursing officers in light of Bourdieu
Source: Rev Esc Enferm USP. 2025 Nov 28;59:e20250234. doi: 10.1590/1980-220X-REEUSP-2025-0234en (PMC12679713; doi:10.1590/1980-220X-REEUSP-2025-0234en)
Supplement: Translation

# Translation: Enfermagem Militar no Exército Brasileiro (1992–2020): perspectivas de oficiais de enfermagem à luz de Bourdieu*

**DOI:** 10.1590/1980-220X-REEUSP-2025-0234pt

**Authors:** Fabrícia Conceição de Carvalho, Maria Itayra Padilha, Fernanda Batista Oliveira Santos, Paulo Joaquim Pina Queirós, Vanessa Ribeiro Neves

**Affiliations:** 1 Exército Brasileiro, Hospital Militar de Área de São Paulo, São Paulo, SP, Brasil.; 2 Universidade Federal Santa Catarina, Departamento de Enfermagem e do Programa de Pós-Graduação em Enfermagem, Florianópolis, SC, Brasil.; 3 Universidade Federal de Minas Gerais, Departamento de Enfermagem Básica, Belo Horizonte, MG, Brasil.; 4 Escola Superior de Enfermagem, Departamento de Enfermagem Fundamental, Coimbra, Portugal.; 5 Universidade Federal de São Paulo, Departamento de Serviços de Administração em saúde e em Enfermagem, São Paulo, SP, Brasil.

**Keywords:** História da Enfermagem, Enfermagem, Militares, Enfermagem Militar, Serviços de Saúde Militar

## Abstract

**Objetivo:**

Analisar as perspectivas de oficiais de Enfermagem acerca da Enfermagem Militar no Exército Brasileiro à luz de Bourdieu, no período de 1992 a 2020.

**Método:**

Estudo de interesse histórico-social, com abordagem qualitativa da História Oral Temática. Foram entrevistados 15 oficiais de Enfermagem que atuaram nos serviços de saúde ou no Fundo de Saúde do Exército Brasileiro. Os dados foram transcritos, transcriados, categorizados, o conteúdo analisado segundo Minayo, e os achados interpretados à luz da Teoria do Mundo Social de Bourdieu.

**Resultados:**

Identificaram-se a categoria “Enfermagem e Exército Brasileiro: o encontro de hierarquizações” e as subcategorias “Valores militares”, “Gênero nas relações militares hierarquizadas” e “Do ser civil para o ser militar”. Na perspectiva dos participantes, a Enfermagem Militar apresenta influências dos capitais militar, gênero e intelectual de Enfermagem para sua identidade profissional.

**Conclusão:**

A trajetória da Enfermagem Militar mantida na atualidade pelos oficiais de Enfermagem do Exército Brasileiro oportuniza perspectivas influenciadas pelo volume de capital nas relações militares hierarquizadas assim como no lucro simbólico da Enfermagem Militar.

## INTRODUÇÃO

A guerra da Crimeia (1853–1856) foi palco para a consolidação da existência da Enfermagem Moderna, que teve a enfermeira Florence Nightingale como protagonista da transformação do cuidado, a partir de seu legado nos hospitais de campanha. Esta transformação possibilitou a disseminação de valores presentes na prática militar do cenário de atuação de Nightingale e sua equipe para a prática de Enfermagem: a hierarquia e a disciplina^(1)^. A Enfermagem e a instituição militar têm uma aproximação histórica, cuja ambiência é um local de prática contemporânea da Enfermagem no mundo e no Brasil^(2)^. Assim, a Enfermagem em contextos de guerra especializa-se como Enfermagem Militar, que, embora não seja formalmente reconhecida no Brasil, é convencionalmente identificada na prática por seus militares de enfermagem.

A cultura organizacional rígida presente na instituição militar é materializada na divisão de funções para o cumprimento de ordens, ao passo que limita a autonomia individual, pois nenhuma ideia ou sugestão modificadora permanece restrita ao seu detentor, sendo sempre reavaliada pela liderança superior a fim de manter o *status quo* organizacional típico de obediência, disciplina e hierarquia^(3)^. Nesse sentido, durante o trabalho nos hospitais de campanha da Crimeia, mesmo cumprindo estritamente as normas, Nightingale sofreu com essa rigidez hierárquica, atrelada à resistência cultural de sua presença feminina, por parte dos oficiais médicos, pois ela e as demais enfermeiras não podiam estar presentes nos pavilhões em que estes oficiais estivessem. Contudo, à medida em que era fiel na observância de formalidades militares, foi obtendo aceitação nas relações com a equipe médica militar e conquistando popularidade por meio de seu trabalho, que, no aspecto sanitário e militar, contribuiu para a redução da taxa de mortalidade e consequente minimização de baixas dos soldados, favorecendo a permanência da tropa no *front* de batalha^(2)^.

Nesse feito histórico da Enfermagem, é possível identificar fatos que materializam os conceitos de *habitus* e de capital nas relações de poder típicas de ambiente militar sob a ótica do filósofo e sociólogo francês Pierre Bourdieu^(4)^. O *habitus* é uma disposição incorporada que se manifesta no estilo de vida, nas atitudes e na maneira de ser dos indivíduos. No contexto militar, ele reflete a internalização do capital militar, legitimado pelas patentes ou graduações. Esses postos, conforme seu volume e prestígio, representam oportunidades concretas de obtenção de lucros simbólicos, derivados do reconhecimento dentro da hierarquia institucional.

O capital é sinônimo de poder, pois reflete a posição de um agente (indivíduo ou instituição) por suas características no sistema social. Assim, o capital pode ser econômico (recurso financeiro), cultural (conhecimento, crenças e valores), social (representatividade no grupo), dentre outros. O detentor de capitais terá como consequência lucro simbólico e poder nas relações sociais^(5)^.

Nesse sentido, a observância de Florence Nightingale às formalidades presentes na hierarquia militar representa a inculcação de um *habitus* que possibilitou a aquisição de capital necessário ao pertencimento no grupo social militar. Esse capital faz parte do capital cultural militar que, associado às patentes ou graduações militares, aumenta o seu volume para a legitimação do lucro simbólico de reconhecimento nas relações de poder do círculo hierárquico militar. Nightingale, por ser oriunda do meio civil, não detinha o capital militar decorrente das patentes, mas seu *habitus* militar, adquirido e associado ao trabalho na Guerra da Crimeia, formou capitais culturais que lhe trouxeram poder para representatividade e significado na Enfermagem que extrapolou a seara militar, tornando-se modelo de profissionalização do cuidado de Enfermagem em todo o mundo^(6)^. Portanto, o ambiente militar foi para a Enfermagem o que a Enfermagem foi para o ambiente militar: um lucro simbólico conquistado por meio da atuação de enfermeiras em campo de batalha, o que ensejou o encontro de hierarquizações representadas por valores militares comuns às duas profissões, Militar e Enfermagem: obediência, disciplina e hierarquia, que originaram a Enfermagem Militar^(7)^.

A Enfermagem Militar está presente no Exército Brasileiro desde 1944, quando da criação do Quadro de Enfermeiras da Reserva da Força Expedicionária Brasileira (FEB) durante a Segunda Guerra Mundial. Com o fim da guerra, uma lacuna de 12 anos instaurou-se e, a partir de 1957, as enfermeiras da reserva da FEB extinguiriam essa lacuna por conta de sua reinclusão como oficiais da ativa neste mesmo quadro^(8)^. Com a aposentadoria das oficiais enfermeiras, nova lacuna da Enfermagem no Exército surge e mantém-se por 35 anos, sendo rompida, em 1992, quando a Enfermagem voltou para as suas fileiras por meio do Quadro Complementar de Oficiais (QCO), complementado pelo apoio técnico da Enfermagem presente também em outro quadro de oficiais criado em 2018: Quadro de Oficial Técnico-Temporário (OTT)^(9,10)^. Esses quadros são de finalidade administrativa e destinam-se ao apoio em assuntos administrativos-militares às especialidades militares do Exército presentes nas armas, quadros e serviços, como o Serviço de Saúde. Assim, a Enfermagem faz-se presente em quadros administrativos do Exército Brasileiro como auxílio a áreas de interesse da instituição para o combate. A esse respeito, particularmente no Serviço de Saúde, a Enfermagem Militar atua na vertente assistencial como apoio a práticas assistenciais, seja pela atuação direta em unidades clínico-cirúrgicas nos hospitais militares, realizando cuidado de Enfermagem segundo o perfil de clientela, seja pela gestão e gerenciamento do serviço de Enfermagem, seja pela educação permanente voltada aos militares de Enfermagem ou pela educação em saúde a usuários do sistema de saúde militar, seja pela atuação indireta, em setores de apoio administrativo-hospitalar como almoxarifado, laboratórios, seção de licitação e contratos ou ainda em ambulatórios de especialidades médicas. Também atua na gestão de contratos de saúde e na auditoria destes contratos nas fases prospectiva, concorrente e retrospectiva de sua execução.

Ainda naquele serviço, a Enfermagem Militar também atua na vertente operacional, prestando atenção pré-hospitalar e aplicando conhecimentos em suportes básico e avançado de vida. Portanto, a Enfermagem Militar atua no Exército Brasileiro conforme as necessidades e os interesses institucionais da Administração Pública Militar, prestando serviço segundo demandas em saúde (Serviço de Saúde) e demandas administrativas das demais especialidades militares (armas, quadros e serviços), sendo todas cumulativas.

Esse acúmulo pode, por vezes, alienar a identidade profissional da Enfermagem pela sobreposição da perspectiva militar no cumprimento de ordens, o que distancia a Enfermagem de sua essência profissional em saúde e de seu protagonismo no cuidado.

A prática profissional da Enfermagem Militar no Exército Brasileiro apresenta uma realidade que foge à realidade habitual da prática no ambiente civil, onde a Enfermagem atua somente na sua área de formação e de especialidade, o que pode contribuir para uma fragilização da imagem da Enfermagem e para a destituição da representatividade da classe profissional.

Destarte, investigações acerca da Enfermagem Militar são importantes para o conhecimento dessa realidade mediante a identificação das percepções dos militares de Enfermagem que atuam nesta carreira secular, a fim de possibilitar o entendimento dos significados do cuidado de Enfermagem exercido em ambiente militar. Ademais, a Enfermagem Militar é uma área com escassez de produção bibliográfica nacional^(11)^, de modo que estudos como este poderão mitigar essa lacuna e propiciar maior clareza acerca do mundo militar como espaço de exercício profissional da Enfermagem. Pretende-se assim, com este estudo, apresentar a vivência da prática profissional militar sob o olhar dos oficiais de Enfermagem pertencentes ao QCO e ao OTT do Exército Brasileiro de forma que se possa contextualizar com a historicidade vivida por Florence Nightingale em cenário de relações de poder do ambiente militar.

A justificativa para este estudo está presente na contribuição que ele possa trazer para a profissão de Enfermagem Militar no Brasil e no mundo no que tange ao conhecimento da área militar para a prática profissional, que ainda apresenta limites quanto à presença do gênero feminino e que, apesar de ser um campo histórico da presença profissional da Enfermagem, ainda é campo recente e carente de inovação científica em Enfermagem, exigindo-se avanços.

A Enfermagem como profissão predominantemente feminina^(11)^, e que nasce a partir da hierarquização do trabalho, revela, por si só, limites no inter-relacionamento profissional, os quais se tornam aumentados em espaços limítrofes de poder, como o espaço militar, favorecendo a desigualdade e a violência simbólica do gênero, além do enfraquecimento do senso de grupo e da identidade profissional em Enfermagem^(5,11)^.

Este estudo tem por objetivo identificar as percepções dos oficiais de Enfermagem acerca da Enfermagem Militar no Exército Brasileiro no período de 1992 a 2020.

## MÉTODO

Estudo descritivo, de interesse histórico-social, com abordagem qualitativa da História Oral Temática^(12)^. Para a elaboração do método foi seguido o roteiro *Consolidated Criteria for Reporting Qualitative Research* (COREQ)^(13)^.

O recorte temporal desta pesquisa inicia-se em 1992 por ter sido o marco para o reingresso do profissional de Enfermagem como oficial de carreira no Quadro Complementar de Oficiais (QCO), e 2020 como recorte final, devido à entrada dos primeiros oficiais de Enfermagem no Quadro de Oficial Técnico-Temporário (OTT), em 2018, apesar de ter sido criado em 2002 por meio do Decreto nº 4.502^(14)^.

Esta pesquisa foi submetida para apreciação do Comitê de Ética em Pesquisa com Seres Humanos (CEP) da Universidade Federal de São Paulo (UNIFESP) sob o certificado de apresentação (CAAE) 54969421.7.0000.5505 e aprovada pelo Parecer no 5.584.693.

A coleta de dados foi feita por meio de roteiro de entrevista semiestruturada aplicada a participantes identificados como Militares 01 a 15, previamente selecionados com base nos critérios de inclusão definidos para o estudo. O questionário apresenta perguntas de interesse sobre liderança, motivação pela carreira, além de os participantes terem tido a abertura para falarem sobre suas vivências profissionais, cujos dados pertinentes foram considerados no estudo.

Os critérios de inclusão deste estudo foram: oficiais de Enfermagem da ativa ou da reserva remunerada (aposentados R1), membros do QCO, e oficiais de Enfermagem da ativa ou da reserva não-remunerada (temporários licenciados de forma voluntária ou após término de 8 anos de serviço R2) membros do OTT que, no período de 1992 a 2020, tenham ocupado cargo de coordenação/gestão de setor hospitalar do Serviço de Saúde do Exército ou cargo de coordenação/gestão do Fundo de Saúde do Exército. Quanto aos critérios de exclusão: oficiais de Enfermagem da ativa ou da reserva (R1), membros do QCO, oficiais de Enfermagem da ativa ou da reserva (R2), membros do OTT que, no recorte temporal deste estudo, ocuparam apenas cargo de coordenação/gestão de Posto Médico de Guarnição ou de Batalhão Militar. Estes espaços não foram considerados no estudo, pois a atuação do oficial enfermeiro QCO ou OTT é mínima ou inexistente, quanto a atividades de liderança em gestão de saúde ou cuidado direto. Para preservar o anonimato do registro, os militares participantes foram representados pela seguinte codificação: militar 01, militar 02 e assim sucessivamente até o militar 15.

Segundo os dados do Sistema de Saúde do Exército Brasileiro obtidos em 2023^(15)^, o número de enfermeiros do QCO existentes no país totalizava 629, enquanto o número de enfermeiros do OTT não foi divulgado. Essa incompletude de dados, associada à dispersão dos enfermeiros militares que atuam nas 12 Regiões Militares, do norte ao sul do Brasil, dificultou a previsão amostral para envio de convites para a participação no estudo. Dessa forma, a coleta de dados iniciada em 2023 ocorreu da seguinte forma: o canal de contato para envio de texto-convite para participação no estudo foi o de *WhatsApp*, haja vista que os canais de comunicação do Exército Brasileiro são exclusivos para assuntos de interesse do Ministério da Defesa relacionados à defesa do território e a missões humanitárias. Assim, grupos de *WhatsApp* de enfermeiros militares dos quadros QCO e OTT foram notificados para participação voluntária no estudo. Houve também a divulgação do estudo aos oficiais de Enfermagem que atuavam na mesma unidade militar da pesquisadora e aos quais, ao demonstrarem interesse na participação, foi enviado o texto-convite. A pesquisadora principal analisou o perfil profissional dos interessados para verificar se era condizente aos critérios de inclusão do estudo, e excluiu os que não os atendiam. Por sua vez, aos que preencheram critérios de inclusão, foi sugerido o convite de mais oficiais de Enfermagem com quem tivessem contato (bola-de-neve). Esses também tiveram seu perfil analisado quanto aos critérios de inclusão do estudo. Por último, foi enviado o Termo de Consentimento Livre e Esclarecido (TCLE) por e-mail aos participantes eleitos e que, após respondidos e assinados, foram enviados de volta ao e-mail da pesquisadora.

Com os dados coletados, seu processo de transcrição, de transcriação, de categorização e de análise do conteúdo foi conduzido de acordo com o modelo de Minayo^(16)^. Já a interpretação dos dados foi feita segundo a Teoria do Mundo Social de Pierre Bourdieu^(17)^.

Esta pesquisa possui dados oriundos da tese “Identidade e perspectivas da Enfermagem Militar Brasileira: uma análise historiográfica (1992-2020), que pode ser obtida por meio do repositório: https://repositorio.unifesp.br/items/49880e2d-d9d3-4420-a7e6-53b1c60f43fc^1^.

Neste texto, selecionou-se o conteúdo das entrevistas mais significativas para a análise das subcategorias “valores militares”, “gênero nas relações militares hierarquizadas”, “do ser civil para o ser militar” à luz de Pierre Bourdieu, o que culminou na seleção das falas dos militares 02, 03, 04, 06, 08 e 10 dos 15 militares participantes da pesquisa referenciada.

## RESULTADOS

Após o atendimento dos critérios de inclusão, restaram 15 participantes, sendo 11 mulheres e quatro homens. Entre as mulheres, quatro eram oficiais de Enfermagem do QCO, sendo uma oficial R1 e as outras três da ativa, e sete eram oficiais de Enfermagem do OTT: uma da ativa e seis oficiais R2. Entre os homens, um era oficial de Enfermagem do QCO, sendo R1, e os outros três eram oficiais de Enfermagem do OTT: todos da ativa. As idades variaram de 32 a 39 anos para oficiais OTT da ativa e oficiais R2, 33 anos para as oficiais QCO da ativa e 52 a 59 anos para oficiais QCO R1. O tempo de atuação profissional foi de dois a cinco anos para oficiais OTT da ativa e até oito anos para oficiais OTT R2. Para os oficiais do QCO, o tempo de atuação profissional foi de nove anos para oficiais da ativa e de 17 a 33 anos para oficiais R1.

A partir da análise do objetivo e das informações de cada entrevista, foi elaborada a hipótese pertinente constante no Quadro 1. Ao realizar a análise de conteúdo^(16)^, foram identificadas 12 unidades de registro dentro da unidade de contexto: “Capitais da profissão de Enfermagem e da profissão militar”. Nessa unidade de contexto, as três subcategorias, “Valores militares”, “O gênero nas relações militares hierarquizadas” e “Do ser civil para o ser militar”, foram identificadas como significado dos discursos dos participantes, e resultaram na categoria “Enfermagem e Exército Brasileiro: o encontro de hierarquizações”.

**Quadro 1:** Esquematização da hipótese – Capitais. São Paulo, SP, Brasil, 2024.

Hipótese: Capitais			
**Categoria**	Enfermagem e Exército Brasileiro: o encontro de hierarquizações.		
**Subcategorias**	Valores militares	Gênero nas relações militares hierarquizadas	Do ser civil para o ser militar
**Unidade de contexto**	Capitais da profissão de Enfermagem e da profissão militar.		
**Unidades de registro**	Resiliência; Comunicação; Espírito de equipe; Liderança; Conhecimento de Enfermagem; Atualização; Conhecimento Militar; Gênero; Resolução de problemas; Gestão de conflitos; Proatividade; Administração pública.		

***Valores militares***

*Por tudo o que vivi, percebo que, para ser oficial de Enfermagem no Exército Brasileiro, é preciso conhecimento técnico, obediência à hierarquia e à disciplina, saber se posicionar e se comunicar, ter resiliência, paciência, saber trabalhar em equipe e ter humildade para aceitar aquilo que não pode ser mudado.* **(Militar 2).**

*[...] o oficial enfermeiro tem que apresentar competências de: conhecimento técnico na área de atuação, aprimoramento por meio de atualização científica, gerir conflitos, ter autoridade, autonomia, ter escuta ativa, tratar todo mundo com respeito. É preciso ter conduta justa nos relacionamentos interprofissionais tratando com desigualdade os desiguais [...].* (**Militar 8).**

*[...] acredito que a Enfermagem Militar é vista com muita admiração por conta da abnegação da vida pessoal [...] Acredito que a Enfermagem Militar é uma área importante da enfermagem brasileira por ter todas essas vertentes: a da defesa da soberania nacional, do auxílio à população e da prontidão para atuar em diversos locais quando for necessário e designado.* (**Militar 10).**

***O gênero nas relações militares hierarquizadas***

*Eu era a única enfermeira desta unidade e quem fazia delegações a mim era uma Capitão médica, que era a chefe do Posto Médico [...] Quando eu mostrava pra ela as necessidades do setor, quando eu falava pra ela “a gente precisa fazer isso, a gente precisa fazer aquilo”, ela dava pouca importância, ao mesmo tempo que ela queria que eu fizesse mudanças no serviço ela me limitava para executar essas mudanças [...].* **(Militar 3).**

*Nas minhas vivências como oficial enfermeira, pude perceber o espanto e a surpresa quando algumas pessoas conheciam minha rotina em algumas atividades militares, ou quando eu apresentava meu nome de guerra* ^2^ *, cujo sobrenome sugestionava que eu pudesse ser um homem. Alguns chegaram a me questionar na minha capacidade feminina para comandar homens.* **(Militar 4).**

*Eu ingresso como enfermeira ganhando por uma patente, e não por ser mulher porque a gente vê muitos empregos no meio civil que há a desvalorização salarial para a mulher. A Enfermagem por ser uma profissão essencialmente feminina tem disparidade salarial frente à profissão de Medicina que beira quatro ou cinco vezes mais. Então, na carreira militar, isso não ocorre porque a Enfermagem ganhará pela patente [...] Tive desafios no exercício da profissão, mas foi uma excelente experiência [...].* **(Militar 8).**

***Do ser civil para o ser militar***

*Eu me formei em Enfermagem em 2010, logo após trabalhei com Epidemiologia por dois anos e em seguida [...] trabalhei no Hospital Municipal por quatro anos. Eu sempre tive um encantamento pelo Exército [...] me enaltecia ao ver mulheres fardadas, mas não tinha ideia de como ter acesso para trabalho nas Forças Armadas até que uma amiga casada com um militar sinalizou sobre o processo seletivo de 2014, em que fiz e fui aprovada [...] Antes de fazer parte do Exército, eu não sabia como era a Enfermagem Militar [...] Os primeiros anos foram muito bons, consegui desenvolver um bom trabalho, ter muita autonomia nas minhas ações, mas ao longo da carreira fui tendo muita dificuldade [...] em que fui me desmotivando com a perda da autonomia e com a prevalência da parte militar sobre a parte de Enfermagem.* (**Militar 2**).

*Eu me formei na Universidade Federal de São Paulo em 2010. Em 2011, trabalhei no Hospital São Paulo na unidade de cirurgia pediátrica e na unidade de transplante de medula óssea pediátrica no GRAAC. No final de 2014, saí do GRAACC e comecei a trabalhar em uma clínica [...] Eu saí dessa clínica para ingressar às fileiras do Exército em 2018 pelo processo seletivo de oficial técnico-temporário[...] a palavra que eu usaria para definir como é trabalhar no Exército seria desafio [...] é um mundo completamente diferente [...] percebi que o hospital militar não tem regras claras como um hospital civil, o que me fez questionar a minha permanência na instituição e a minha autonomia como enfermeira [...] Além de todas estas dificuldades institucionais à nossa rotina de trabalho, eu tenho uma inquietação sobre o papel do enfermeiro aqui dentro: sempre quando vejo um enfermeiro em funções que não necessárias serem executadas por ele [...] me traz incômodo [...].* (**Militar 6).**

## DISCUSSÃO

A Enfermagem constrói a sua história profissional nos *fronts* de guerra, estabelecendo uma identidade profissional que no campo militar se apropria de uma especificidade quanto a valores militares, vivência de conquistas, avanços e desafios, principalmente acerca da inserção do gênero feminino em relações militares hierarquizadas, além do choque cultural a partir da entrada de um *habitus* profissional civil que tenta se moldar para pertencimento em campo militar^(3,7,17,18)^. Nesse sentido, valores militares, o gênero feminino em relações hierarquizadas e a expertise profissional civil de Enfermagem, adentrando em ambiente militar, serão discutidos para possibilitar o conhecimento e a reflexão acerca da realidade de prática militar de Enfermagem.

A Enfermagem Militar possui a particularidade profissional de uma identidade que considera valores extrínsecos à Enfermagem, que são os militares. Sobre os valores militares, o aprimoramento técnico-profissional esteve presente nos discursos como relevante para a prática militar de Enfermagem. Também foram identificados a fé na missão, que é sinônimo de resiliência frente às adversidades e desafios vividos na carreira militar, e o patriotismo que enaltece o servir à Pátria. O amor à profissão foi percebido nas falas sobre a dedicação e abnegação exigidas na carreira militar. Por sua vez, o espírito de corpo foi identificado quando os depoentes se referiram ao trabalho em equipe como fator a ser considerado pelo oficial de Enfermagem. Estes valores oportunizam a caracterização de capitais simbólicos^(17,18)^ presentes na identidade profissional da Enfermagem Militar. De acordo com Bourdieu^(18)^, o *habitus* prepara o indivíduo para o espaço social dotando-o de habilidades que o caracterizam para o pertencimento no ambiente. Desta forma, quando um profissional de Enfermagem possuidor de *habitus* do ambiente civil de saúde adentra o ambiente militar, ele é preparado para a aquisição de *habitus* militar identificado pelo traje da farda, o rigor do estilo de cabelo e barba, da ausência de adornos, da maquiagem e uso de esmalte discreto para mulheres, também o uso de medalhas quando condecorado por algum feito extraordinário, da entonação da voz para ordens de comando em situações de atividades militares, dos movimentos corporais sincronizados e com tônus muscular nas formaturas militares. Assim, a transição da carreira civil de Enfermagem para a carreira militar promove uma transformação pessoal, que resulta em capital militar associado ao capital intelectual que diferencia a Enfermagem Militar para ganho simbólico no espaço de atuação profissional militar.

Sobre o gênero nas relações militares hierarquizadas, foi perceptível a visão de cada oficial de Enfermagem frente ao gênero feminino no Exército Brasileiro. Particularmente, para oficiais enfermeiras participantes do estudo, a carreira militar oferta melhores condições de trabalho se comparada ao meio civil, quanto à igualdade de exercício profissional e de salário, pois essa equivalência se dá pelo posto hierárquico. Embora haja desigualdade na representação feminina nos altos postos de comando, observa-se que poucas mulheres alcançam o posto de coronel. Além disso, até o momento da realização deste estudo, nenhuma mulher havia ascendido ao posto de general.

Tudo isso externaliza a dificuldade de aceitação da presença feminina pelo grupo militar, o que conota uma resistência cultural ainda existente tal qual a ocorrida no campo de batalha com Florence Nightingale em 1853^(2)^.

Houve ainda relatos de violência simbólica entre as próprias mulheres, quando em um mesmo espaço profissional-militar, seja como líder ou como membro da equipe, não há apoio feminino neste ambiente. A esse respeito, é importante discutir o conceito de violência simbólica sob a ótica de Bourdieu. Esta é uma forma de violência invisível que se impõe em uma relação do tipo subjugação-submissão, na qual o reconhecimento e a cumplicidade na violência são componentes presentes da relação dominador-dominado. A linguagem, os gestos, a exclusão social, os símbolos e formas de comunicação ou de conhecimento são exemplos de dominação simbólica que se instauram por intermédio de um processo sustentado pela existência e pelo reforço de pensamentos e predisposições alinhados às estruturas impostas^(19)^.

O Estado, como exemplo de instituto organizativo hegemônico, além da família e da igreja, utiliza agentes como Forças Militares para estabelecer a conformidade social e moral. Por sua vez, esses agentes dotados de relação organizacional hierarquizante apropriam-se dos meios, dos materiais e dos simbolismos que garantem a conformidade da lógica e da moral às maneiras de compreender e construir o mundo aos interesses dominantes, e isso resultará na violência simbólica tão naturalizada e intrínseca. O gênero feminino, como grupo sócio historicamente subjugado pela dominação masculina, perpetua a relação desigual de gênero em espaços de poder densamente hierarquizados como Forças de Militares^(19,20)^. Logo, a ocupação de liderança e representatividade em alto escalão de poder militar, hoje, limita-se majoritariamente ao grupo masculino, sendo que nos postos militares de coronel encontram-se baixas representações femininas e, de generais, por ora, inexistentes para mulheres no Exército Brasileiro.

Ademais, o capital militar atrelado à posição na hierarquia favorece a disputa de poder no campo, em que o volume deste capital influencia para legitimação dos agentes. Associado a isso, nota-se que o capital simbólico de gênero, ainda não validado institucionalmente para pertencimento e aceitação pelo grupo militar masculino, exprime percepção social desfavorável entre as mulheres por não deterem este capital e a validação de sua presença^(5,19-25)^. Logo, é por meio do capital militar que a mulher tenta marcar a sua presença no campo militar e atingir poder.

Na visão de um oficial homem entrevistado, a mulher tem avançado em sua presença no cenário militar, desde a atuação nas guerras, elevando a representatividade feminina nos espaços militares masculinos.

De fato, o gênero feminino obteve avanços quando ousou ocupar espaços tradicionalmente masculinos, como o espaço militar, favorecendo não só para efeito simbólico de ressignificação social da imagem da mulher como ganho simbólico para espaço profissional conquistado por profissões femininas como a Enfermagem^(3,5,6)^. Contudo, ainda há desafios a serem enfrentados quanto à resistência cultural de sua presença para ascensão profissional de determinados cargos militares, que, no caso do Exército Brasileiro, se relaciona com cargos de alta liderança militar de oficial general, pois ainda não estão disponíveis para mulheres, tampouco para a Enfermagem.

O capital simbólico^(18)^ de gênero surge como volume adicional ao capital militar nas relações militares hierarquizadas em que, especificamente no Exército Brasileiro, o capital representado na profissão ainda repele forças atinentes a outros agentes e seus capitais, como o masculino, por ser minoria quantitativa e qualitativa no círculo social militar^(5,20–25)^.

Na lógica de relações de poder^(17,18)^ existentes em qualquer grupo social, no grupo militar o gênero feminino presente na Enfermagem Militar enfrenta lutas que se intensificam pelos capitais simbólicos de gênero e intelectuais de Enfermagem, cuja legitimação é enfraquecida no ciclo hierárquico militar, que é densamente masculino e médico-centrado. Assim, a Enfermagem Militar nas relações militares hierarquizadas apresenta similaridade com a violência simbólica vivida por Florence Nightingale na Guerra da Crimeia, no século 19, embora seja necessário relativizar esta semelhança, considerando que, naquela época, não era esperado das mulheres atuação profissional qualificada e muito menos alguma posição de destaque hierárquico no âmbito militar^(1,2)^.

Atualmente, a Enfermagem Militar é majoritariamente feminina (capital simbólico) e também subvalorizada, pois está em quadros administrativos e não no Serviço de Saúde, não tendo poder para validação institucional e possibilidade de transformação desta sub-representação por meio de uma liderança de Enfermagem Militar em alta cúpula de oficiais do Serviço de Saúde: posto hierárquico de oficial general.

Existe uma dissociação entre Enfermagem Assistencial e Enfermagem Administrativa, sendo que há uma priorização para a prática militar, promovendo o foco em tarefas administrativas e atividades militares, havendo a alienação de sua identidade completa como profissional em saúde. A consequência é a invalidação institucional como profissão da saúde para pertencimento ao Serviço de Saúde das Forças Armadas além de uma identidade profissional militar, o que favorece a perpetuação do modelo de atenção vigente e a dominação hegemônica médica^(23)^.

Sobre a trajetória profissional dos oficiais de Enfermagem, a maioria veio da sociedade civil com um *habitus* diverso do militar, o que gera muitos conflitos quando no desempenho da Enfermagem Militar^(3)^. À exceção de alguns militares participantes do estudo, que já possuíam *habitus* militar ao adentrarem as fileiras do Exército para a naturalização das especificidades da prática de Enfermagem Militar, os demais entrevistados apresentaram frustração explícita ou implícita, observada na expressão facial durante as entrevistas, frente à prática profissional de Enfermagem Militar deexigir abnegação de sua identidade profissional em Enfermagem para cumprimento de ordens militares fundamentadas pelos princípios organizacionais de hierarquia e disciplina^(19)^.

Sobre o poder simbólico na prática de Enfermagem Militar do Exército Brasileiro, a hierarquia militar valida-o para reconhecimento institucional da Enfermagem como agente militar, ao passo que fragiliza a identidade profissional da Enfermagem pela predominância da identidade militar por meio de determinação à Enfermagem na realização de ações não relacionadas com a prática de saúde, mas com a prática de trabalho militar e que inviabilizam a imagem da Enfermagem como profissão do cuidado^(9,10)^.

Pelo exposto, identificou-se que da Enfermagem Civil para a Enfermagem Militar acontece uma transformação cultural de inculcação do *habitus* militar para que se forme o oficial de Enfermagem, o que, para a maioria dos oficiais participantes deste estudo, é motivo de estranhamento e frustração da prática profissional militar, com a imposição deste *habitus* à identidade profissional de Enfermagem.

Assim, os oficiais de Enfermagem, no cumprimento de ordens militares que distanciam o oficial da prática profissional de Enfermagem — pois essas ordens representam o *habitus* militar —, têm seu poder simbólico na Enfermagem limitado e sua identidade profissional de Enfermagem Militar fragilizada. Isso faz com que as relações de poder existentes no grupo social militar apresentem forças contrárias impeditivas à existência da Enfermagem no Exército Brasileiro, revelando a perpetuação da resistência à presença da Enfermagem no cenário militar no Brasil, desde a presença das oficiais-enfermeiras da Força Expedicionária Brasileira.

Assim, este estudo apresentou como limitação o acesso presencial aos participantes para coleta de dados, uma vez que todos se encontravam dispersos territorialmente, o que justificou a coleta de informações para participação voluntária, após divulgação do texto-convite nos grupos de *WhatsApp*, por meio de e-mail enviado, *Google Forms* para participação dos convidados dos participantes (bola-de-neve), uso da plataforma *Zoom* para a realização das entrevistas.

A contribuição deste estudo é ser fonte de dados sobre Enfermagem Militar no Brasil e no mundo, por ser uma área que carece de mais publicações para preenchimento de lacunas bibliográficas sobre a identidade profissional da Enfermagem Militar. Ainda, instiga a reflexão e o pensamento crítico sobre o grupo profissional militar de Enfermagem e os capitais como cerne para o poder simbólico da Enfermagem no cenário militar, favorecendo o esclarecimento da lógica de poder que perpetua a barreira de invalidação da Enfermagem como profissão do cuidado no Exército Brasileiro.

## CONCLUSÃO

Apesar de a Enfermagem ser uma profissão que tem a história narrada por meio da história militar, que, por sua vez, está presente na história da profissão de Enfermagem desde 1853, a Enfermagem Militar enfrenta desafios de reconhecimento e de pertencimento nas Forças Armadas. No Brasil, por ser o Exército uma instituição tradicional que preza por uma cultura de valoração dos feitos heroicos e de manutenção de crenças que moldam a prática profissional militar, isso dificulta transformações de modelos organizacionais de trabalho, que no caso da saúde militar possui modelo materializado pela preservação da atenção tradicionalmente centrada no oficial-médico.

Como o modelo de atenção em saúde militar do Exército Brasileiro é medicalocêntrico e focado em condutas intervencionistas, em uma perspectiva pragmática típica do âmbito militar, a Enfermagem Militar ainda é desconsiderada no seu papel profissional de saúde. Portanto, o poder simbólico da Enfermagem Militar está atrelado à patente hierárquica e não ao seu conhecimento em saúde; quando há uma sobreposição da patente (capital militar) em uma relação em que há uma capitã-enfermeira e um tenente-médico, à capitã será dada voz e legitimidade devido ao capital militar conferir-lhe poder nas Forças Armadas. Contudo, quando, na relação militar, há um mesmo capital em disputa para poder, tenente-enfermeira *versus* tenente-médico, ou a predominância do capital militar-médico na relação capitão-médico *versus* tenente-enfermeira, haverá a destituição do poder simbólico da Enfermagem para serem validados o capital militar e o capital médico reconhecidos no campo militar.

Os resultados deste estudo revelaram que os pilares militares de hierarquia e disciplina, valores da profissão militar, pautaram a profissão de Enfermagem a partir da guerra na Crimeia, bem como a profissão de Enfermagem Militar, o que possibilitou sua identidade profissional no campo militar. Essa possui o capital simbólico do gênero, o capital militar e o capital intelectual de Enfermagem, que poderão ou não representar o poder que legitime uma identidade segundo a percepção e atuação dos oficiais de Enfermagem no círculo hierárquico militar.

A trajetória da Enfermagem Militar mantida na atualidade pelos oficiais de Enfermagem do Exército Brasileiro oportuniza perspectivas da prática que são influenciadas pelo peso e volume de capital nas relações militares hierarquizadas e que validam a identidade profissional da Enfermagem Militar no Exército Brasileiro. A esse respeito, pode-se inferir que de 1992 a 2020, quando foi permitida a atuação da Enfermagem no Exército Brasileiro, por meio do Quadro Complementar de Oficiais e do Quadro de Oficial Técnico-Temporário, houve ganho simbólico para a Enfermagem e para a mulher brasileira que, em espaço de poder militar, conquistaram igualdade salarial e igualdade de exercício profissional, ainda que permaneça, na atualidade, a desigualdade da ocupação de altos cargos de liderança.

Como efeito simbólico da presença da Enfermagem no Exército Brasileiro, possibilitou-se uma identidade militar à profissão de Enfermagem atuante neste cenário, e contribuiu-se para uma nova especialidade: a Enfermagem Militar.

## Data Availability

Os dados que dão suporte aos resultados deste estudo estão disponíveis no repositório institucional da UNIFESP, no seguinte link: https://repositorio.unifesp.br/items/49880e2d-d9d3-4420-a7e6-53b1c60f43fc.

# Translation: Enfermería militar en el Ejército brasileño (1992–2020): perspectivas de los oficiales de enfermería a la luz de Bourdieu*

**DOI:** 10.1590/1980-220X-REEUSP-2025-0234es

**Authors:** Fabrícia Conceição de Carvalho, Maria Itayra Padilha, Fernanda Batista Oliveira Santos, Paulo Joaquim Pina Queirós, Vanessa Ribeiro Neves

**Affiliations:** 1 Exército Brasileiro, Hospital Militar de Área de São Paulo, São Paulo, SP, Brasil.; 2 Universidade Federal Santa Catarina, Departamento de Enfermagem e do Programa de Pós-Graduação em Enfermagem, Florianópolis, SC, Brasil.; 3 Universidade Federal de Minas Gerais, Departamento de Enfermagem Básica, Belo Horizonte, MG, Brasil.; 4 Escola Superior de Enfermagem, Departamento de Enfermagem Fundamental, Coimbra, Portugal.; 5 Universidade Federal de São Paulo, Departamento de Serviços de Administração em saúde e em Enfermagem, São Paulo, SP, Brasil.

**Keywords:** Historia de la Enfermería, Enfermería, Personal Militar, Enfermería Militar, Servicios de Salud Militares

## Abstract

**Objetivo:**

Analizar las perspectivas de los oficiales de Enfermería sobre la Enfermería Militar en el Ejército Brasileño a la luz de Bourdieu, de 1992 a 2020.

**Método:**

Estudio de interés histórico-social, con un enfoque cualitativo de la Historia Oral Temática. Se entrevistaron quince oficiales de enfermería que trabajaban en servicios de salud o en la Caja de Salud del Ejército Brasileño. Los datos fueron transcritos, transcreados, categorizados, el contenido analizado según Minayo y los hallazgos interpretados a la luz de la Teoría del Mundo Social de Bourdieu.

**Resultados:**

Se identificaron la categoría “Enfermería y Ejército Brasileño: el encuentro de jerarquías” y las subcategorías “Valores militares”, “Género en las relaciones jerárquicas militares” y “De ser civil a ser militar”. Desde la perspectiva de los participantes, la Enfermería Militar presenta influencias de los capitales militar, de género e intelectual de la Enfermería en su identidad profesional.

**Conclusión:**

La trayectoria de la Enfermería Militar mantenida hoy por oficiales de Enfermería del Ejército Brasileño ofrece perspectivas influenciadas por el volumen de capital en las relaciones militares jerárquicas, así como en el lucro simbólico de la Enfermería Militar.

## INTRODUCCIÓN

La guerra de Crimea (1853–1856) fue el escenario de la consolidación de la existencia de la enfermería moderna, que tuvo a la enfermera Florence Nightingale como protagonista de la transformación de la atención, a partir de su legado en los hospitales de campaña. Esta transformación permitió la difusión de los valores presentes en la práctica militar del escenario de actuación de Nightingale y su equipo a la práctica de la enfermería: la jerarquía y la disciplina^(1)^. La enfermería y la institución militar tienen una aproximación histórica, cuyo ambiente es un lugar de práctica contemporánea de la enfermería en el mundo y en Brasil^(2)^. Así, la enfermería en contextos de guerra se especializa como enfermería militar, que, aunque no está formalmente reconocida en Brasil, se identifica convencionalmente en la práctica por sus militares de enfermería.

La rígida cultura organizativa presente en la institución militar se materializa en la división de funciones para el cumplimiento de órdenes, al tiempo que limita la autonomía individual, ya que ninguna idea o sugerencia modificadora permanece restringida a su titular, sino que siempre es reevaluada por los superiores con el fin de mantener el statu quo organizativo típico de obediencia, disciplina y jerarquía^(3)^. En este sentido, durante su trabajo en los hospitales de campaña de Crimea, incluso cumpliendo estrictamente las normas, Nightingale sufrió esta rigidez jerárquica, unida a la resistencia cultural a su presencia femenina por parte de los oficiales médicos, ya que ella y las demás enfermeras no podían estar presentes en las salas en las que se encontraban estos oficiales. Sin embargo, a medida que se mostraba fiel en el cumplimiento de las formalidades militares, fue ganando aceptación en las relaciones con el equipo médico militar y popularidad a través de su trabajo, que, en el aspecto sanitario y militar, contribuyó a la reducción de la tasa de mortalidad y la consiguiente minimización de bajas de los soldados, favoreciendo la permanencia de las tropas en el frente de batalla^(2)^.

En este hecho histórico de la enfermería, es posible identificar hechos que materializan los conceptos de *habitus* y capital en las relaciones de poder típicas del entorno militar desde la perspectiva del filósofo y sociólogo francés Pierre Bourdieu^(4)^. El *habitus* es una disposición incorporada que se manifiesta en el estilo de vida, las actitudes y la forma de ser de los individuos. En el contexto militar, refleja la internalización del capital militar, legitimado por los rangos o grados. Estos puestos, según su volumen y prestigio, representan oportunidades concretas de obtener beneficios simbólicos, derivados del reconocimiento dentro de la jerarquía institucional.

El capital es sinónimo de poder, ya que refleja la posición de un agente (individuo o institución) por sus características en el sistema social. Así, el capital puede ser económico (recursos financieros), cultural (conocimientos, creencias y valores), social (representatividad en el grupo), entre otros. El poseedor de capital obtendrá como consecuencia ganancias simbólicas y poder en las relaciones sociales^(5)^.

En este sentido, el cumplimiento por parte de Florence Nightingale de las formalidades presentes en la jerarquía militar representa la inculcación de un habitus que le permitió adquirir el capital necesario para pertenecer al grupo social militar. Este capital forma parte del capital cultural militar que, asociado a los rangos o grados militares, aumenta su volumen para legitimar el beneficio simbólico del reconocimiento en las relaciones de poder del círculo jerárquico militar. Nightingale, al provenir del ámbito civil, no poseía el capital militar derivado de los rangos, pero su habitus militar, adquirido y asociado al trabajo en la Guerra de Crimea, formó capitales culturales que le dieron poder de representatividad y significado en la enfermería que extrapoló el ámbito militar, convirtiéndose en un modelo de profesionalización de la atención de enfermería en todo el mundo^(6)^. Por lo tanto, el entorno militar fue para la enfermería lo que la enfermería fue para el entorno militar: un beneficio simbólico conquistado a través de la actuación de las enfermeras en el campo de batalla, lo que dio lugar al encuentro de jerarquías representadas por valores militares comunes a ambas profesiones, la militar y la enfermería: obediencia, disciplina y jerarquía, que dieron origen a la enfermería militar^(7)^.

La enfermería militar está presente en el Ejército brasileño desde 1944, cuando se creó el Cuadro de Enfermeras de la Reserva de la Fuerza Expedicionaria Brasileña (FEB) durante la Segunda Guerra Mundial. Con el fin de la guerra, se produjo un vacío de 12 años y, a partir de 1957, las enfermeras de la reserva de la FEB llenaron ese vacío al reincorporarse como oficiales en activo en este mismo cuadro^(8)^. Con la jubilación de las oficiales enfermeras, surge una nueva brecha en la enfermería del Ejército que se mantiene durante 35 años, hasta que se rompe en 1992, cuando la enfermería regresó a sus filas a través del Cuadro Complementario de Oficiales (CCO), complementado por el apoyo técnico de la enfermería también presente en otro cuadro de oficiales creado en 2018: el Cuadro de Oficiales Técnicos Temporales (OTT)^(9,10)^. Estos cuadros tienen fines administrativos y están destinados a prestar apoyo en asuntos administrativos-militares a las especialidades militares del Ejército presentes en las armas, cuadros y servicios, como el Servicio de Salud. Así, la enfermería está presente en los cuadros administrativos del Ejército Brasileño como ayuda en áreas de interés de la institución para el combate. En este sentido, particularmente en el Servicio de Salud, la Enfermería Militar actúa en el ámbito asistencial como apoyo a las prácticas asistenciales, ya sea mediante la actuación directa en unidades clínico-quirúrgicas en hospitales militares, realizando cuidados de enfermería según el perfil de la clientela, ya sea mediante la gestión y administración del servicio de enfermería, ya sea mediante la educación permanente dirigida al personal militar de enfermería o la educación en salud a los usuarios del sistema de salud militar, ya sea mediante la actuación indirecta, en sectores de apoyo administrativo-hospitalario como almacenes, laboratorios, sección de licitaciones y contratos o incluso en consultorios de especialidades médicas. También actúa en la gestión de contratos de salud y en la auditoría de estos contratos en las fases prospectiva, concurrente y retrospectiva de su ejecución.

Además, en ese servicio, la Enfermería Militar también actúa en el ámbito operativo, prestando atención prehospitalaria y aplicando conocimientos en soporte vital básico y avanzado. Por lo tanto, la Enfermería Militar actúa en el Ejército Brasileño de acuerdo con las necesidades e intereses institucionales de la Administración Pública Militar, prestando servicios según las demandas en salud (Servicio de Salud) y las demandas administrativas de las demás especialidades militares (armas, cuadros y servicios), todas ellas acumulativas.

Esta acumulación puede, en ocasiones, alienar la identidad profesional de la enfermería por la superposición de la perspectiva militar en el cumplimiento de órdenes, lo que aleja a la enfermería de su esencia profesional en salud y de su protagonismo en la atención.

La práctica profesional de la enfermería militar en el Ejército brasileño presenta una realidad que se aleja de la realidad habitual de la práctica en el ámbito civil, donde la enfermería actúa únicamente en su área de formación y especialidad, lo que puede contribuir a debilitar la imagen de la enfermería y a destituir la representatividad de la clase profesional.

Por lo tanto, las investigaciones sobre la enfermería militar son importantes para conocer esta realidad mediante la identificación de las percepciones de los militares de enfermería que trabajan en esta carrera secular, con el fin de permitir la comprensión de los significados de la atención de enfermería ejercida en el entorno militar. Además, la enfermería militar es un área con escasa producción bibliográfica nacional^(11)^, por lo que estudios como este pueden mitigar esta laguna y aportar mayor claridad sobre el mundo militar como espacio de ejercicio profesional de la enfermería. Así, con este estudio se pretende presentar la experiencia de la práctica profesional militar desde la perspectiva de los oficiales de enfermería pertenecientes al CCO y al OTT del Ejército Brasileño, de manera que se pueda contextualizar con la historicidad vivida por Florence Nightingale en un escenario de relaciones de poder del entorno militar.

La justificación de este estudio radica en la contribución que puede aportar a la profesión de enfermería militar en Brasil y en el mundo en lo que respecta al conocimiento del ámbito militar para la práctica profesional, que aún presenta límites en cuanto a la presencia del género femenino y que, a pesar de ser un campo histórico de la presencia profesional de la enfermería, sigue siendo un campo reciente y carente de innovación científica en enfermería, lo que exige avances.

La enfermería, como profesión predominantemente femenina^(11)^, que además nace de la jerarquización del trabajo, revela, por sí sola, límites en la interrelación profesional, que se acentúan en espacios limítrofes de poder, como el espacio militar, favoreciendo la desigualdad y la violencia simbólica de género, además del debilitamiento del sentido de grupo y de la identidad profesional en enfermería^(5,11)^.

El objetivo de este estudio es identificar las percepciones de los oficiales de enfermería sobre la enfermería militar en el Ejército Brasileño en el período de 1992 a 2020.

## MÉTODO

Estudio descriptivo, de interés histórico-social, con un enfoque cualitativo de la Historia Oral Temática^(12)^. Para la elaboración del método se siguió la guía *Consolidated Criteria for Reporting Qualitative Research* (COREQ)^(13)^.

El periodo temporal de esta investigación comienza en 1992, ya que fue el hito para el reingreso del profesional de enfermería como oficial de carrera en el Cuadro Complementario de Oficiales (CCO), y termina en 2020, debido a la entrada de los primeros oficiales de enfermería en el Cuadro de Oficiales Técnicos Temporales (OTT) en 2018, a pesar de haber sido creado en 2002 mediante el Decreto n.º 4.502^(14)^.

Esta investigación fue sometida a la consideración del Comité de Ética en Investigación con Seres Humanos (CEP) de la Universidad Federal de São Paulo (UNIFESP) bajo el certificado de presentación (CAAE) 54969421.7.0000.5505 y aprobada por el Dictamen n.º 5.584.693.

La recolección de datos se realizó mediante un guion de entrevista semiestructurada aplicada a participantes identificados como Militares 01 a 15, previamente seleccionados en función de los criterios de inclusión definidos para el estudio. El cuestionario presenta preguntas de interés sobre liderazgo, motivación por la carrera, además de que los participantes tuvieron la oportunidad de hablar sobre sus experiencias profesionales, cuyos datos pertinentes se consideraron en el estudio.

Los criterios de inclusión de este estudio fueron: oficiales de enfermería en activo o en la reserva remunerada (jubilados R1), miembros del CCO y oficiales de enfermería en activo o en la reserva no remunerada (licenciados temporales de forma voluntaria o tras cumplir 8 años de servicio R2), miembros del OTT que, en el periodo comprendido entre 1992 y 2020, hayan ocupado un cargo de coordinación/gestión del sector hospitalario del Servicio de Salud del Ejército o un cargo de coordinación/gestión del Fondo de Salud del Ejército. En cuanto a los criterios de exclusión: oficiales de enfermería en activo o en la reserva (R1), miembros del CCO, oficiales de enfermería en activo o en la reserva (R2), miembros del OTT que, en el periodo de tiempo de este estudio, ocuparon únicamente cargos de coordinación/gestión del Puesto Médico de Guarnición o del Batallón Militar. Estos espacios no se consideraron en el estudio, ya que la actuación del oficial de enfermería del CCO o del OTT es mínima o inexistente en lo que respecta a las actividades de liderazgo en la gestión de la salud o la atención directa. Para preservar el anonimato del registro, los militares participantes fueron representados mediante la siguiente codificación: militar 01, militar 02 y así sucesivamente hasta el militar 15.

Según los datos del Sistema de Salud del Ejército Brasileño obtenidos en 2023^(15)^, el número de enfermeros del CCO existentes en el país ascendía a 629, mientras que el número de enfermeros del OTT no se divulgó. Esta incompletitud de datos, asociada a la dispersión de los enfermeros militares que actúan en las 12 Regiones Militares, de norte a sur de Brasil, dificultó la previsión muestral para el envío de invitaciones para participar en el estudio. De este modo, la recolección de datos iniciada en 2023 se llevó a cabo de la siguiente manera: el canal de contacto para enviar el texto de la invitación a participar en el estudio fue *WhatsApp*, dado que los canales de comunicación del Ejército Brasileño son exclusivos para asuntos de interés del Ministerio de Defensa relacionados con la defensa del territorio y las misiones humanitarias. Así, se notificó a grupos de WhatsApp de enfermeros militares de las plantillas CCO y OTT para que participaran voluntariamente en el estudio. También se dio a conocer el estudio a los oficiales de enfermería que trabajaban en la misma unidad militar que la investigadora y a quienes, al mostrar interés en participar, se les envió la invitación. La investigadora principal analizó el perfil profesional de los interesados para verificar si se ajustaba a los criterios de inclusión del estudio y excluyó a los que no los cumplían. A su vez, a los que cumplían los criterios de inclusión se les sugirió que invitaran a más oficiales de enfermería con los que tuvieran contacto (efecto bola de nieve). También se analizó el perfil de estos últimos en relación con los criterios de inclusión del estudio. Por último, se envió por correo electrónico el Formulario de Consentimiento Libre y Esclarecido (FCLE) a los participantes seleccionados y, una vez respondido y firmado, se devolvió al correo electrónico de la investigadora.

Con los datos recopilados, el proceso de transcripción, transcreción, categorización y análisis del contenido se llevó a cabo de acuerdo con el modelo de Minayo^(16)^. La interpretación de los datos se realizó según la Teoría del Mundo Social de Pierre Bourdieu^(17)^.

Esta investigación cuenta con datos procedentes de la tesis “Identidad y perspectivas de la Enfermería Militar Brasileña: un análisis historiográfico (1992-2020)”, que puede obtenerse a través del repositorio: https://repositorio.unifesp.br/items/49880e2d-d9d3-4420-a7e6-53b1c60f43fc ^1^.

En este texto, se seleccionó el contenido de las entrevistas más significativas para el análisis de las subcategorías “valores militares”, “género en las relaciones militares jerárquicas”, “del ser civil al ser militar” a la luz de Pierre Bourdieu, lo que culminó en la selección de las declaraciones de los militares 02, 03, 04, 06, 08 y 10 de los 15 militares participantes en la investigación mencionada.

## RESULTADOS

Tras cumplir los criterios de inclusión, quedaron 15 participantes, 11 mujeres y cuatro hombres. Entre las mujeres, cuatro eran oficiales de enfermería del CCO, una de ellas oficial R1 y las otras tres en activo, y siete eran oficiales de enfermería del OTT: una en activo y seis oficiales R2. Entre los hombres, uno era oficial de enfermería del CCO, siendo R1, y los otros tres eran oficiales de enfermería del OTT: todos en servicio activo. Las edades variaron de 32 a 39 años para los oficiales del OTT en servicio activo y los oficiales R2, 33 años para las oficiales del CCO en servicio activo y de 52 a 59 años para las oficiales del CCO R1. La antigüedad profesional era de dos a cinco años para los oficiales OTT en activo y de hasta ocho años para los oficiales OTT R2. En el caso de los oficiales del CCO, la antigüedad profesional era de nueve años para los oficiales en activo y de 17 a 33 años para los oficiales R1.

A partir del análisis del objetivo y de la información de cada entrevista, se elaboró la hipótesis pertinente que figura en el Cuadro 1. Al realizar el análisis de contenido^(16)^, se identificaron 12 unidades de registro dentro de la unidad de contexto: “Capitales de la profesión de enfermería y de la profesión militar”. En esta unidad de contexto, las tres subcategorías, “Valores militares”, “El género en las relaciones militares jerárquicas” y “Del ser civil al ser militar”, se identificaron como significado de los discursos de los participantes y dieron lugar a la categoría “Enfermería y Ejército Brasileño: el encuentro de jerarquizaciones”.

**Cuadro 1:** Esquematización de la hipótesis – Capitales. São Paulo, SP, Brasil, 2024.

Hipótesis: Capitales			
**Categoría**	Enfermería y el Ejército Brasileño: el encuentro de jerarquizaciones.		
**Subcategorías**	Valores militares	Género en las relaciones militares jerarquizadas	Del ser civil al ser militar
**Unidad de contexto**	Capitales de la profesión de enfermería y de la profesión militar.		
**Unidades de registro**	Resiliencia; Comunicación; Espíritu de equipo; Liderazgo; Conocimientos de enfermería; Actualización; Conocimientos militares; Género; Resolución de problemas; Gestión de conflictos; Proactividad; Administración pública.		

***Valores militares***

*Por todo lo que he vivido, me doy cuenta de que, para ser oficial de enfermería en el Ejército brasileño, se necesita conocimiento técnico, obediencia a la jerarquía y a la disciplina, saber posicionarse y comunicarse, tener resiliencia, paciencia, saber trabajar en equipo y tener la humildad de aceptar lo que no se puede cambiar.* ***(Militar 2).***

*[...] el oficial de enfermería debe presentar las siguientes competencias: conocimientos técnicos en el área de actuación, perfeccionamiento mediante actualización científica, gestión de conflictos, autoridad, autonomía, escucha activa, trato respetuoso a todas las personas. Es necesario tener una conducta justa en las relaciones interprofesionales, tratando con desigualdad a los desiguales [...]. (**Militar 8).***

*[...] creo que la enfermería militar es vista con mucha admiración debido a la abnegación de la vida personal [...] Creo que la enfermería militar es un área importante de la enfermería brasileña por tener todas estas vertientes: la defensa de la soberanía nacional, la ayuda a la población y la prontitud para actuar en diversos lugares cuando sea necesario y se le asigne. (**Militar 10).***

***El género en las relaciones jerárquicas militares***

*Yo era la única enfermera de esta unidad y quien me delegaba tareas era una capitana médica, que era la jefa del puesto médico [...] Cuando le mostraba las necesidades del sector, cuando le decía “tenemos que hacer esto, tenemos que hacer aquello”, ella le daba poca importancia, al mismo tiempo que quería que yo hiciera cambios en el servicio, me limitaba para ejecutar esos cambios [...].* **(Militar 3).**

*En mis experiencias como oficial de enfermería, pude percibir el asombro y la sorpresa cuando algunas personas conocían mi rutina en algunas actividades militares, o cuando presentaba mi nombre de guerra, cuyo apellido sugería que yo podía ser un hombre. Algunos incluso cuestionaron mi capacidad femenina para comandar a los hombres.* **(Militar 4).**

*Entro como enfermera ganando por un rango, y no por ser mujer, porque vemos muchos empleos en el ámbito civil en los que se infravalora el salario de las mujeres. La enfermería, al ser una profesión esencialmente femenina, tiene una disparidad salarial frente a la profesión de medicina, que es casi cuatro o cinco veces mayor. Sin embargo, en la carrera militar esto no ocurre porque la enfermería gana por el rango [...] Tuve retos en el ejercicio de la profesión, pero fue una experiencia excelente [...].* ***(*Militar 8).**

***Del ser civil al ser militar***

*Me gradué en Enfermería en 2010, luego trabajé en Epidemiología durante dos años y después [...] trabajé en el Hospital Municipal durante cuatro años. Siempre me ha fascinado el Ejército [...] me emocionaba ver a las mujeres con uniforme, pero no tenía ni idea de cómo acceder al trabajo en las Fuerzas Armadas hasta que una amiga casada con un militar me habló del proceso de selección de 2014, al que me presenté y aprobé [...] Antes de formar parte del Ejército, no sabía cómo era la enfermería militar [...] Los primeros años fueron muy buenos, logré desarrollar un buen trabajo, tener mucha autonomía en mis acciones, pero a lo largo de mi carrera tuve muchas dificultades [...] y me desmotivé por la pérdida de autonomía y por el predominio de la parte militar sobre la parte de enfermería.* (**Militar 2**).

*Me gradué en la Universidad Federal de São Paulo en 2010. En 2011, trabajé en el Hospital São Paulo en la unidad de cirugía pediátrica y en la unidad de trasplante de médula ósea pediátrica en el GRAAC. A finales de 2014, dejé el GRAACC y empecé a trabajar en una clínica [...] Dejé esa clínica para alistarme en el Ejército en 2018 mediante el proceso de selección de oficial técnico temporal [...] la palabra que utilizaría para definir cómo es trabajar en el Ejército sería “desafío” [...] es un mundo completamente diferente [...] me di cuenta de que el hospital militar no tiene reglas claras como un hospital civil, lo que me hizo cuestionar mi permanencia en la institución y mi autonomía como enfermera [...] Además de todas estas dificultades institucionales en nuestra rutina de trabajo, me preocupa el papel del enfermero aquí: siempre que veo a un enfermero realizando funciones que no le corresponden [...] me molesta [...].* (**Militar 6).**

## DISCUSIÓN

La enfermería construye su historia profesional en los frentes de guerra, estableciendo una identidad profesional que, en el ámbito militar, se apropia de una especificidad en cuanto a valores militares, vivencias de conquistas, avances y retos, principalmente en lo que se refiere a la inserción del género femenino en las relaciones militares jerárquicas, además del choque cultural a partir de la entrada de un *habitus* profesional civil que intenta amoldarse para pertenecer al campo militar^(3,7,17,18)^. En este sentido, se discutirán los valores militares, el género femenino en relaciones jerárquicas y la experiencia profesional civil de la enfermería, al entrar en el ámbito militar, para permitir el conocimiento y la reflexión sobre la realidad de la práctica militar de la enfermería.

La Enfermería Militar tiene la particularidad profesional de una identidad que considera valores extrínsecos a la enfermería, que son los militares. En cuanto a los valores militares, el perfeccionamiento técnico-profesional estuvo presente en los discursos como relevante para la práctica militar de la enfermería. También se identificaron la fe en la misión, que es sinónimo de resiliencia frente a las adversidades y los retos vividos en la carrera militar, y el patriotismo que ensalza el servicio a la patria. El amor por la profesión se percibió en los comentarios sobre la dedicación y la abnegación que exige la carrera militar. A su vez, se identificó el espíritu de cuerpo cuando los entrevistados se refirieron al trabajo en equipo como un factor a tener en cuenta por el oficial de enfermería. Estos valores permiten caracterizar los capitales simbólicos^(17,18)^ presentes en la identidad profesional de la enfermería militar. Según Bourdieu^(18)^, el *habitus* prepara al individuo para el espacio social dotándolo de habilidades que lo caracterizan para pertenecer al entorno. De esta manera, cuando un profesional de enfermería que posee el *habitus* del entorno civil de la salud entra en el entorno militar, está preparado para adquirir el *habitus* militar identificado por el uniforme, el rigor del estilo de cabello y barba, la ausencia de adornos, el maquillaje y el uso de esmalte discreto para las mujeres, también el uso de medallas cuando se le condecora por algún hecho extraordinario, la entonación de la voz para las órdenes de mando en situaciones de actividades militares, los movimientos corporales sincronizados y con tono muscular en las formaciones militares. Así, la transición de la carrera civil de enfermería a la carrera militar promueve una transformación personal, que da lugar a un capital militar asociado al capital intelectual que diferencia a la enfermería militar para obtener un beneficio simbólico en el ámbito de la actuación profesional militar.

En cuanto al género en las relaciones jerárquicas militares, fue perceptible la visión de cada oficial de enfermería frente al género femenino en el Ejército Brasileño. En particular, para las oficiales enfermeras que participaron en el estudio, la carrera militar ofrece mejores condiciones de trabajo en comparación con el ámbito civil, en cuanto a la igualdad en el ejercicio profesional y el salario, ya que esta equivalencia se da por el puesto jerárquico. Aunque existe desigualdad en la representación femenina en los altos puestos de mando, se observa que pocas mujeres alcanzan el puesto de coronel. Además, hasta el momento de la realización de este estudio, ninguna mujer había ascendido al puesto de general.

Todo ello pone de manifiesto la dificultad de aceptación de la presencia femenina por parte del grupo militar, lo que denota una resistencia cultural aún existente, similar a la que se produjo en el campo de batalla con Florence Nightingale en 1853^(2)^.

También ha habido denuncias de violencia simbólica entre las propias mujeres, cuando en un mismo espacio profesional-militar, ya sea como líder o como miembro del equipo, no hay apoyo femenino en este entorno. A este respecto, es importante discutir el concepto de violencia simbólica desde la perspectiva de Bourdieu. Se trata de una forma de violencia invisible que se impone en una relación de tipo subyugación-sumisión, en la que el reconocimiento y la complicidad en la violencia son componentes presentes en la relación dominador-dominado. El lenguaje, los gestos, la exclusión social, los símbolos y las formas de comunicación o de conocimiento son ejemplos de dominación simbólica que se instauran a través de un proceso sustentado por la existencia y el refuerzo de pensamientos y predisposiciones alineados con las estructuras impuestas^(19)^.

El Estado, como ejemplo de institución organizativa hegemónica, además de la familia y la iglesia, utiliza agentes como las Fuerzas Armadas para establecer la conformidad social y moral. A su vez, estos agentes dotados de una relación organizativa jerárquica se apropian de los medios, los materiales y los simbolismos que garantizan la conformidad de la lógica y la moral con las formas de comprender y construir el mundo según los intereses dominantes, lo que da lugar a una violencia simbólica tan naturalizada e intrínseca. El género femenino, como grupo socio-históricamente sometido a la dominación masculina, perpetúa la relación de género desigual en espacios de poder densamente jerarquizados como las Fuerzas Armadas^(19,20)^. Por lo tanto, la ocupación de puestos de liderazgo y representatividad en las altas esferas del poder militar se limita hoy en día mayoritariamente al grupo masculino, ya que en los puestos militares de coronel hay una escasa representación femenina y, por el momento, no hay mujeres generales en el Ejército brasileño.

Además, el capital militar vinculado a la posición en la jerarquía favorece la disputa por el poder en el campo, donde el volumen de este capital influye en la legitimación de los agentes. A esto se suma que el capital simbólico de género, aún no validado institucionalmente para la pertenencia y aceptación por parte del grupo militar masculino, expresa una percepción social desfavorable entre las mujeres por no poseer este capital y la validación de su presencia^(5,19,20,21,22,23,24,25)^. Por lo tanto, es a través del capital militar que la mujer intenta marcar su presencia en el campo militar y alcanzar el poder.

En opinión de un oficial (hombre) entrevistado, la mujer ha avanzado en su presencia en el ámbito militar, desde su actuación en las guerras, elevando la representatividad femenina en los espacios militares masculinos.

De hecho, el género femenino ha logrado avances al atreverse a ocupar espacios tradicionalmente masculinos, como el espacio militar, lo que ha favorecido no solo el efecto simbólico de la resignificación social de la imagen de la mujer, sino también la ganancia simbólica del espacio profesional conquistado por profesiones femeninas como la enfermería^(3,5,6)^. Sin embargo, aún quedan retos por afrontar en cuanto a la resistencia cultural a su presencia para el ascenso profesional a determinados puestos militares que, en el caso del Ejército brasileño, están relacionados con puestos de alta dirección militar de oficial general, ya que aún no están disponibles para las mujeres, ni tampoco para la enfermería.

El capital simbólico^(18)^ de género surge como un volumen adicional al capital militar en las relaciones militares jerárquicas en las que, específicamente en el Ejército Brasileño, el capital representado en la profesión aún repele fuerzas relacionadas con otros agentes y sus capitales, como el masculino, por ser una minoría cuantitativa y cualitativa en el círculo social militar^(5,20,21,22,23,24,25)^.

En la lógica de las relaciones de poder^(17,18)^ existentes en cualquier grupo social, en el grupo militar, el género femenino presente en la enfermería militar se enfrenta a luchas que se intensifican por los capitales simbólicos de género e intelectuales de la enfermería, cuya legitimación se ve debilitada en el ciclo jerárquico militar, que es densamente masculino y centrado en la medicina. Así, la enfermería militar en las relaciones militares jerárquicas presenta similitudes con la violencia simbólica que vivió Florence Nightingale en la Guerra de Crimea, en el siglo XIX, aunque es necesario relativizar esta similitud, teniendo en cuenta que, en aquella época, no se esperaba de las mujeres una actuación profesional cualificada y mucho menos una posición destacada en la jerarquía militar^(1,2)^.

En la actualidad, la enfermería militar es mayoritariamente femenina (capital simbólico) y también está infravalorada, ya que se encuentra en puestos administrativos y no en el Servicio de Salud, sin poder para la validación institucional y sin posibilidad de transformar esta subrepresentación mediante un liderazgo de enfermería militar en la alta cúpula de oficiales del Servicio de Salud: puesto jerárquico de oficial general.

Existe una disociación entre la enfermería asistencial y la enfermería administrativa, y se da prioridad a la práctica militar, promoviendo el enfoque en tareas administrativas y actividades militares, lo que supone la alienación de su identidad completa como profesional en salud. La consecuencia es la invalidación institucional como profesión de la salud para pertenecer al Servicio de Salud de las Fuerzas Armadas, además de una identidad profesional militar, lo que favorece la perpetuación del modelo de atención vigente y el dominio médico hegemónico^(23)^.

En cuanto a la trayectoria profesional de los oficiales de enfermería, la mayoría procedía de la sociedad civil con un habitus diferente al militar, lo que genera muchos conflictos en el desempeño de la enfermería militar^(3)^. A excepción de algunos militares participantes en el estudio, que ya poseían un habitus militar al ingresar en las filas del Ejército para la naturalización de las especificidades de la práctica de la enfermería militar, los demás entrevistados mostraron una frustración explícita o implícita, observada en la expresión facial durante las entrevistas, ante la práctica profesional de la enfermería militar que exige la abnegación de su identidad profesional en enfermería para cumplir con las órdenes militares basadas en los principios organizativos de jerarquía y disciplina^(19)^.

En relación al poder simbólico en la práctica de la enfermería militar del Ejército brasileño, es validado por la jerarquía militar para el reconocimiento institucional de la enfermería como agente militar, mientras que debilita la identidad profesional de la enfermería por el predominio de la identidad militar a través de la determinación de la enfermería en la realización de acciones no relacionadas con la práctica de la salud, sino con la práctica del trabajo militar y que inviabilizan la imagen de la enfermería como profesión de cuidado^(9,10)^.

Por lo expuesto, se identificó que de la Enfermería Civil a la Enfermería Militar se produce una transformación cultural de inculcación del habitus militar para formar al oficial de Enfermería, lo que, para la mayoría de los oficiales participantes en este estudio, es motivo de extrañeza y frustración de la práctica profesional militar, con la imposición de este habitus a la identidad profesional de la Enfermería.

Así, los oficiales de enfermería, al cumplir órdenes militares que los alejan de la práctica profesional de la enfermería —ya que estas órdenes representan el habitus militar—, ven limitado su poder simbólico en la enfermería y debilitada su identidad profesional de enfermería militar. Esto hace que las relaciones de poder existentes en el grupo social militar presenten fuerzas contrarias que dificultan la existencia de la enfermería en el Ejército Brasil, revelando la perpetuación de la resistencia a la presencia de la enfermería en el escenario militar en Brasil, desde la presencia de las oficiales enfermeras de la Fuerza Expedicionaria Brasileña.

Así, este estudio presentó como limitación el acceso presencial a los participantes para la recopilación de datos, ya que todos se encontraban dispersos territorialmente, lo que justificó la recopilación de información para la participación voluntaria, tras la difusión del texto de invitación en los grupos de WhatsApp, mediante el envío de correos electrónicos, Google Forms para la participación de los invitados de los participantes (bola de nieve) y el uso de la plataforma Zoom para realizar las entrevistas.

La contribución de este estudio es ser una fuente de datos sobre la enfermería militar en Brasil y en el mundo, ya que es un área que carece de más publicaciones para llenar las lagunas bibliográficas sobre la identidad profesional de la enfermería militar. Además, estimula la reflexión y el pensamiento crítico sobre el grupo profesional militar de enfermería y los capitales como núcleo del poder simbólico de la enfermería en el ámbito militar, favoreciendo la clarificación de la lógica de poder que perpetúa la barrera de invalidación de la enfermería como profesión de cuidado en el Ejército Brasileño.

## CONCLUSIÓN

A pesar de que la enfermería es una profesión cuya historia se narra a través de la historia militar, que a su vez está presente en la historia de la profesión de enfermería desde 1853, la enfermería militar se enfrenta a retos de reconocimiento y pertenencia en las Fuerzas Armadas. En Brasil, dado que el Ejército es una institución tradicional que valora una cultura de reconocimiento de los actos heroicos y de mantenimiento de las creencias que moldean la práctica profesional militar, esto dificulta las transformaciones de los modelos organizativos de trabajo, que en el caso de la salud militar se materializan en la preservación de la atención tradicionalmente centrada en el oficial médico.

Dado que el modelo de atención sanitaria militar del Ejército brasileño es médico-centrado y se centra en conductas intervencionistas, desde una perspectiva pragmática típica del ámbito militar, la Enfermería Militar sigue siendo menospreciada en su función profesional de la salud. Por lo tanto, el poder simbólico de la Enfermería Militar está vinculado al rango jerárquico y no a sus conocimientos en materia de salud; cuando se produce una superposición de rangos (capital militar) en una relación en la que hay una capitana enfermera y un teniente médico, a la capitana se le dará voz y legitimidad debido al capital militar que le confiere poder en las Fuerzas Armadas. Sin embargo, cuando en la relación militar hay un mismo capital en disputa por el poder, teniente enfermera versus teniente médico, o el predominio del capital militar-médico en la relación capitán médico versus teniente enfermera, se producirá la destitución del poder simbólico de la enfermería para validar el capital militar y el capital médico reconocidos en el ámbito militar.

Los resultados de este estudio revelaron que los pilares militares de jerarquía y disciplina, valores de la profesión militar, marcaron la profesión de enfermería a partir de la guerra de Crimea, así como la profesión de enfermería militar, lo que permitió su identidad profesional en el campo militar. Esta cuenta con el capital simbólico del género, el capital militar y el capital intelectual de la enfermería, que pueden o no representar el poder que legitima una identidad según la percepción y la actuación de los oficiales de enfermería en el círculo jerárquico militar.

La trayectoria de la enfermería militar que mantienen actualmente los oficiales de enfermería del Ejército brasileño ofrece perspectivas de la práctica que están influenciadas por el peso y el volumen del capital en las relaciones jerárquicas militares y que validan la identidad profesional de la enfermería militar en el Ejército brasileño. A este respecto, se puede inferir que, entre 1992 y 2020, cuando se permitió la actuación de la enfermería en el Ejército Brasileño, a través del Cuadro Complementario de Oficiales y del Cuadro de Oficiales Técnicos Temporales, hubo una ganancia simbólica para la enfermería y para las mujeres brasileñas que, en el ámbito del poder militar, lograron la igualdad salarial y la igualdad en el ejercicio profesional, aunque actualmente persiste la desigualdad en el ocupar altos cargos de liderazgo.

Como efecto simbólico de la presencia de la enfermería en el Ejército brasileño, se logró una identidad militar para la profesión de enfermería que actúa en este ámbito y se contribuyó a una nueva especialidad: la Enfermería Militar.

## Data Availability

Los datos que respaldan los resultados de este estudio están disponibles en el repositorio institucional de la UNIFESP, en el siguiente enlace: https://repositorio.unifesp.br/items/49880e2d-d9d3-4420-a7e6-53b1c60f43fc.
